# Redox-Mediated Incorporation of Sulfated Cellulose Nanocrystals into PVAc Latex: A Comprehensive Structure–Property-Durability Investigations

**DOI:** 10.1021/acsomega.6c06928

**Published:** 2026-10-07

**Authors:** Shahab Nasr, Pooria Khalili, Juliana Aristéia de Lima, Fathi Gouda, Gunnar Westman, Ivo Heinmaa, Mikael Skrifvars

**Affiliations:** † Swedish Centre for Resource Recovery, 1802 University of Borås, 501 90 Borås, Sweden; ‡ Department of Polymer, Fiber and Composite, RISE Research Institutes of Sweden, 501 15 Borås, Sweden; § Chalmers University of Technology, 412 96 Gothenburg, Sweden; ∥ 113902 National Institute of Chemical Physics and Biophysics, Tallinn 12618, Estonia

## Abstract

The role of a redox initiator, in combination with a nonionic surfactant and poly­(ethylene glycol) methacrylate (MPEG), was investigated in the synthesis of nanocomposites of sulfated cellulose nanocrystals and vinyl acetate (VAc) via semibatch emulsion polymerization. This study also explored the effects of heat treatment, including prolonged exposure and annealing. An increase in the CNCs concentration led to aggregation and the formation of larger, irregularly shaped polymer particles. Microscopy analysis indicated increased surface roughness at higher CNCs loadings. The reduction in the optical transparency of the nanocomposites was mitigated by annealing. The contact angle of the untreated samples increased after thermal treatment, particularly at higher CNCs concentrations, by approximately 160%. Water uptake was minimized at 0.6 wt %, and the lowest permeability was observed at 1.9 wt % of CNCs/VAc. The glass transition temperature of the nanocomposites was significantly reduced by 8 °C only under the combined effect of higher CNCs loading and annealing. Annealing resulted in an increase in the storage modulus, particularly for nanocomposites with 1.9 wt % of CNCs/VAc. Creep decreased significantly at high CNCs concentrations after annealing. The elastic modulus and ultimate strength increased with the CNCs content, achieving enhancements of 356% and 80% postannealing for 1.9 and 2.5 wt % of CNCs/VAc, respectively. Finally, the thermal stability of the nanocomposites improved, although variations in the CNCs concentration had minimal additional impact. These results provide insights into how CNC incorporation into poly­(vinyl acetate) latex affects the mechanical, barrier, and surface properties of water-based coatings in applications such as paper coating.

## Graphical abstract

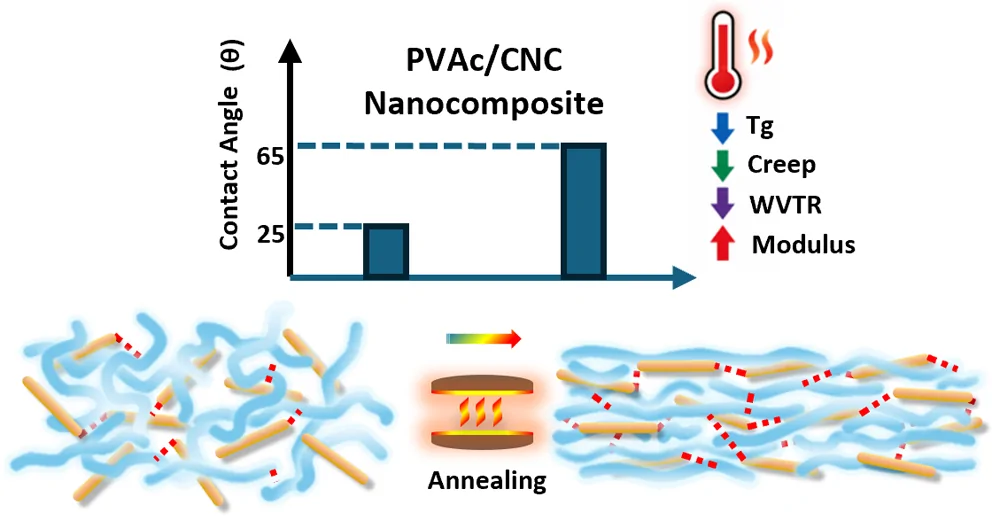

## 1 Introduction

Water-based latex polymers are widely used in various applications such as coatings and adhesives. The synthesis of nanocomposite latex using environmentally sustainable processes and materials presents additional advantages. Vinyl acetate is a biodegradable material that can be degraded under anaerobic and aerobic conditions. It is unlikely to bioaccumulate in organisms and is not classified as persistent, bioaccumulative, or toxic within the European Union.^1^ Among initiators, persulfate is distinguished by its rapid degradation, absence of bioaccumulation, and minimal environmental volatility.^2^ Sulfated cellulose nanocrystals (CNCs) offer several advantages, including biobased origin, low toxicity, and surface modifiability through hydroxyl groups.^3,4^

Among the various techniques for incorporating CNCs into a matrix, water-based emulsion polymerization offers notable benefits. The hydroxyl groups present on the CNCs surfaces can serve as active sites for initiating polymer growth or forming various chemical bonds with the polymer chains, thereby enhancing CNCs dispersion within the polymer matrix.^5^

Application of thermal initiators, such as ammonium persulfate, was previously reported for grafting from CNCs particles in a study by Tang, Lee.^6^ Numerous other studies have documented the use of redox initiators to create initiation sites on the surface of CNCs in aqueous environments.^7–10^

Mabrouk, Dufresne^3^ reported that during in situ aqueous polymerization, CNCs preferentially remained in the continuous phase rather than adsorbing onto the polymer particle surfaces. They also observed that the addition of CNCs had no significant influence on the particle size under surfactant-free conditions. They hypothesized that the presence of unbound CNCs in the aqueous phase hindered polymer–particle coalescence by forming a network. Their research demonstrated that the integration of MPEG (Mn = 375 g·mol^–1^) positively influenced the adsorption of CNCs onto polymer particles, resulting in the formation of a dense CNCs layer on the polymer particles. This, in turn, leads to a reduction in the size of the polymer particles through Pickering effect.^3^

Some studies have indicated that the incorporation of specific polymers, such as poly­(vinyl alcohol) or poly­(ethylene glycol), facilitates the integration of CNCs with a polymer. However, their impact on mechanical reinforcement is typically moderate.^11^ Mabrouk, Dufresne^3^ investigated the effect of adding MPEG on the viscosity of nanocomposites, where MPEG caused a reduction in the viscosity of the system.

The negative surface charge of CNCs particles allows the stability of the CNCs aqueous suspension with an electrical double layer (EDL) surrounding each particle.^12^ The anionic sulfate half-ester groups (–SO_3_^–^) covalently attached to the cellulose surface are responsible for the formation of the electrical double layer on the CNCs particles. It has been observed that aqueous CNCs solutions display shear-thinning properties, typical of non-Newtonian fluids, where their viscosity diminishes as the shear rate increases.^12^ The viscosity of negatively charged sulfated CNCs suspensions is controlled by the ionic strength of the aqueous phase and CNCs surface charge density, in addition to the CNCs concentration. Therefore, at low shear rates, the EDL dominates the viscosity of the suspension, making it difficult for the particles to pass over each other.^12^ An increase in the particle concentration that overlaps with the electrical double layer may result in the formation of a stiff gel. In the presence of salts or electrolytes, the surface charge of the particles is shielded, leading to a reduction in the EDL thickness and subsequent compression of the EDL.^12^ The incorporation of CNCs into the polymerization process of vinyl acetate resulted in a nanocomposite exhibiting a gel-like appearance at rest, as corroborated by Mabrouk, Dufresne.^3^ In their study of nanocomposite latex, they found that the stiffness of the network in dispersion increased with increasing CNCs content and decreased because of the presence of MPEG. This finding indicates that in the absence of MPEG, most CNCs remain dispersed in water without adhering to the polymer particles, thereby facilitating network formation through hydrogen bonding.

While our previous research established the feasibility of synthesizing stable PVAc/CNCs nanocomposite lattices via semibatch emulsion polymerization, further refinement of the formulation and process parameters was required.^13^ This study examined the effects of polymerization temperature, inclusion of MPEG as a component, variations in the initiator system, increased surfactant concentration, and reduction of poly­(vinyl alcohol) (PVOH) content on the polymerization process. The utilization of poly­(vinyl alcohol) (PVOH) as a protective colloid is limited by its insufficient resistance to water, thermal stability, and creep.^14,15^ The substitution of poly­(vinyl alcohol) with surfactants can be achieved by considering the type and concentration of the surfactants. Typically, surfactants are employed in the range of 1–6 wt % relative to the weight of the monomer. In terms of particle size control, surfactants demonstrate superior efficacy compared to protective colloids.^16^ In this study, a nonionic surfactant was selected to minimize effects associated with ionic interactions and changes in the electrostatic environment of the negatively charged CNCs.^12^

Among the various parameters that influence the characteristics of the final polymer, reaction temperature is a critical factor. It can significantly impact the Brownian motion, the morphology of the latex, and the rate of polymerization.^17,18^

The rate of radical formation depends on various parameters, such as temperature, pH, ammonium persulfate concentration, and ionic strength of the solution. Temperature is the primary factor influencing the rate of thermal initiator decomposition, as elevated temperatures promote the formation of a greater number of radicals.^19^

In contrast to conventional thermal initiators, the redox system is activated at lower temperatures through the interaction between its components, specifically the oxidizing and reducing agents. This situation facilitates a more efficient polymerization process.^16,20^ Lower temperatures enhance the control of the reactions, either at initiation stage or during the post polymerization process.^17,21,22^

The incorporation of sodium metabisulfite into a redox system may also serve as an effective oxygen scavenger. In situations where oxygen is either present or produced as a byproduct of certain side reactions, sodium metabisulfite can effectively remove it ([Disp-formula eq1]). The presence of oxygen can result in complications, such as the formation of microbubbles on CNCs or oxygen-associated inhibitory effects.

1a $${2HSO}_{3}^{-}+O_{2}\to {2SO}_{4}^{2-}+{2H}^{+}$$

Oxygen can function as a radical scavenger, leading to the formation of a proxy radical that is typically significantly less reactive than the initial radicals.^23,24^ This process contributes to the inhibition or retardation of polymerization ([Disp-formula eq2])­

2a $$R^{•}+O_{2}\to {ROO}^{•}$$

Accordingly, this study was designed to determine whether the simultaneous modification of the initiation pathway and stabilization system can improve the synthesis and end-use performance of PVAc/CNCs nanocomposites prepared via aqueous semibatch emulsion polymerization. A redox initiator system was employed instead of the thermal ammonium persulfate initiation used in our previous study,^13^ enabling a direct comparison between the redox- and thermally initiated synthesis routes. Simultaneously, the formulation was adjusted by reducing the PVOH content and increasing the emulsifier level, based on the premise that these changes may affect particle formation, colloidal stability, film formation, and the final nanocomposite structure. It was hypothesized that these modifications would influence not only the morphology of the latex particles and films but also the thermal, mechanical, viscoelastic, and barrier properties of the resulting materials. To test this, the morphology of the samples was examined using optical microscopy and scanning electron microscopy, and the structural features were further investigated using NMR. The thermal endurance and dimensional stability of the films were assessed for both the initially prepared films and those following annealing, as well as after extended thermal treatment for durations of 500 and 1000 h above the glass transition temperature. The mechanical and viscoelastic behaviors were assessed using tensile testing, dynamic mechanical analysis, and creep measurements, whereas the barrier performance was evaluated using water vapor transmission rate and swelling tests. The thermal degradation behavior was investigated using thermogravimetric analysis. Finally, statistical analyses were performed to determine the significance of the differences arising from the formulation changes and thermal treatments.

## 2 Materials and Methods

### 2.1 Materials

Cellulose nanocrystals (CNCs) containing sulfate half-ester groups and sodium counterions, produced from northern bleached softwood kraft pulp, were supplied by CelluForce Inc. (Montreal, Quebec, Canada). The CNCs were delivered in a spray-dried form with a sulfur content of 0.89 wt %. (equivalent to 0.0462 OSO_3_^–^ per anhydro glucose unit) and a reported aspect ratio of 20.

Vinyl acetate (VAc) (CAS.108–05–4) (≥99%, Sigma-Aldrich) containing 3–20 ppm hydroquinone was used as received. Ammonium peroxydisulfate (CAS.7727–54–0) ((NH_4_)_2_ S_2_ O_8_, Alfa Aesar, Kandel, Germany) and sodium bicarbonate (CAS.144–55–8) (Sigma-Aldrich) were used as received. Sodium disulfite (CAS.7681–57–4) (Na_2_S_2_O_5_, *M*_w_ = 190.11) was obtained from Avantor. The nonionic emulsifier Emulsogen LCN287 (HLB = 17.5), a fatty alcohol ethoxylated with 28 mol of ethylene oxide, was supplied by Clariant International Ltd., Muttenz, Switzerland. Partially saponified poly­(vinyl alcohol) (PVOH 8–88; degree of hydrolysis 86.7 to 88.7%, with an approximate degree of blockiness of 50%) was sourced from Kuraray Europe GmbH, Germany. Poly­(ethylene glycol) methacrylate (MPEG) (CAS.25736–86–1) was obtained from Sigma-Aldrich with an average Mn of 360 g/mol and 500–800 ppm MEHQ as an inhibitor. Demineralized water was used throughout this study and was prepared using Milli-Q (IQ 7000, 18.2 mΩ·cm @25 °C). All materials were used as received.

#### 2.1.1 Emulsion Polymerization for Synthesis of Nanocomposite Latex

The emulsion polymerization of vinyl acetate was conducted in the presence of cellulose nanocrystals, following the formulations specified in Table 1. Polymerization was conducted in a five-neck, jacketed glass reactor (2 L capacity) equipped with a vertical condenser and a two-stage, top-mounted pitched blade stirrer. CNCs were magnetically dispersed in approximately 80% of the total water volume and subsequently sonicated in an ice bath for 15 min at 80% amplitude using a probe sonicator (S24d7 prob, Up400 St; Hielscher). Sonication disrupted CNC aggregates and improved their dispersion, thereby reducing the size of the scattering entities and consequently decreasing the turbidity of the suspension.^25^ The remaining water was used to dissolve PVA, LCN287, and MPEG, which were subsequently added to the CNCs dispersions. Five minutes before initiating polymerization, the reactor was purged with nitrogen to remove the oxygen. Polymerization was initiated by increasing the temperature to 60 °C and adding 40 wt % of the initiator. The redox initiator solutions (APS/Na_2_S_2_O_5_/H_2_O) were mixed using a static mixer before entering the reactor, along with 3 wt % of VA monomer. After stirring for 15 min, the remaining VA monomer and redox solutions were continuously fed into the reactor for 5.5 h.

**Table 1:** Recipe for the Emulsion Polymerization of Vinyl Acetate in the Presence of CNCs via Redox Initiator (Amounts in g)

	Formulation (g)				
Component	PVAc reference	PC3	PC6	PC9	PC12
Vinyl acetate (VAc)	465	465	465	465	465
CNCs, based on CNCs/VAc (wt %)	-	0.6	1.2	1.9	2.5
Ammonium peroxydisulfate	0.7	0.7	0.7	0.7	0.7
Sodium bicarbonate	0.7	0.7	0.7	0.7	0.7
Sodium disulphite	0.1	0.1	0.1	0.1	0.1
Poly(vinyl alcohol) 8–88	2	2	2	2	2
LCN287	16.3	16.3	16.3	16.3	16.3
Poly(ethylene glycol) methacrylate	1	1	1	1	1
Distilled water	477	477	477	477	477
Total weight of ingredients (g)	962.9	965.9	968.9	971.9	974.9

#### 2.1.2 Preparation of Nanocomposite Films

The nanocomposite films were prepared by casting the dispersions over Teflon paper using a film applicator set to a wet film thickness of 1000 μm, followed by drying in a conventional oven at 60 °C for 2 h. The resulting films were carefully peeled off the Teflon substrate and stored in sealed plastic bags within closed containers to prevent moisture absorption before further analysis.

#### 2.1.3 Thermal Treatment of Nanocomposite Films

Thermal treatments were conducted under two distinct conditions in an oxygenated environment. The thermal-oxidative process was performed in accordance with ASTM D7444–18a. The films were kept in a conventional oven at 60 °C and 9% relative humidity for 500 and 1000 h.

The annealing of the films was conducted in an oven under controlled conditions. The samples were heated at a rate of 15 °C/min, increasing from ambient temperature to 200 °C. Subsequently, they were maintained at 200 °C for 5 min before being allowed to cool to room temperature. To ensure the absence of any alterations in the chemical structure or evidence of degradation, the annealed samples were subjected to further analysis using FTIR spectroscopy. The results indicated no degradation or changes in the chemical structure.

The degree and nature of these changes are influenced by the duration and temperature of exposure. Samples that were not subjected to heat treatment were termed untreated samples.

### 2.2 Characterization Methods

#### 2.2.1 Scanning Electron Microscopy

Freeze-dried latex dispersions were characterized by scanning electron microscopy (SEM) 244 using a Zeiss Supra 40 VP SEM with a secondary electron detector (SED) at an acceleration rate of 7 kV. Subsequently, the cryofracture surfaces were covered with a 15 nm gold layer.

#### 2.2.2 ^13^C MAS NMR Spectra of CNC-PVAc Samples

^13^C CP MAS NMR spectra were recorded on a Bruker AVANCE-II spectrometer in a 14.1 T magnetic field using a home-built MAS probe for 25 mm × 4 mm Si_3_N_4_ rotors. In all experiments, the spinning speed was set to 13.5 kHz. The NMR spectra were recorded using an ordinary cross-polarization (CP) pulse sequence, where the duration of the ramped polarization transfer pulse was 2 ms, the relaxation delay between the excitation times was 5 s, and the signal acquisition was performed in the presence of a strong (90 kHz) decoupling pulse.

The NMR spectra were normalized by dividing the signal intensity by the number of accumulations and the mass of the samples. The normalized spectra were used to estimate the actual PVAc/CNCs ratio. All the samples were originally white powders. At 13.5 kHz fast sample spinning, the temperature of the spinning rotor was 312 K. Therefore, all samples inside the spinning rotors became solid cylinders.

#### 2.2.3 Polarization Optical Microscopy

An optical polarized light microscope (Nikon LV100ND), equipped with a Nikon DS-Fi3 camera, was employed to examine the sample films. Initially, a 200 μm wet film of the sample was applied onto a glass slide using a film applicator and subsequently dried in a ventilation oven at 60 °C for 2 min. Image acquisition and analysis were conducted using NIS-Elements software (version 5.41.01). The images obtained were used to analyze the nanocomposite latex surface. The laboratory conditions during microscopic analysis were maintained at 26 °C and 17% relative humidity.

Following the initial imaging of the films, the samples were annealed to evaluate the effect of thermal treatment on their microstructures and surface morphologies. This process was conducted using a Linkam heating stage connected to the microscope at a heating rate of 15 °C/min from room temperature to 200 °C. The samples were then held at 200 °C for 5 min before being cooled back to room temperature. The positions of the samples remained unchanged before and after annealing to facilitate comparison.

To facilitate a direct comparison of the surface profiles, each z-profile was vertically aligned by subtracting the reference height from the respective scan, and consistent *y*-axis limits were applied to all profiles.

#### 2.2.4 Optical Transmittance Measurements

The optical transmittance of the samples was assessed in the wavelength range of 400–700 nm using a GENESYS 180 spectrophotometer (Thermo Fisher Scientific; software version: 3.3.0.6) in the transmittance mode. Each formulation was applied to glass slides using a 200 μm wet-film applicator and subsequently dried in a ventilated oven at 60 °C for 2 min. An uncoated glass slide was used as a blank reference. Following the initial optical transmittance measurements, the samples were annealed, as described in Section 2.1.3. The annealed samples were subsequently reassessed for their optical transmittance. The values reported are the means of three replicates for each formulation.

#### 2.2.5 Water Contact Angle

The wettability of the samples was assessed using the sessile drop method with an optical contact angle measurement system (OneAttension; Biolin Scientific). A distilled water droplet (3 μL) was carefully placed on the sample surface using an automated dosing system. The droplet profile was recorded for 10 s immediately after deposition. The samples were prepared by applying the formulations onto clean glass slides using a film applicator to achieve a wet thickness of 200 μm. The coated slides were subsequently dried in a conventional oven at 65 °C for 2 min. For the annealed samples, the procedure outlined in Section 2.1.3 was employed.

Each sample underwent six measurements at distinct surface locations for every assessment. The reported values represent the mean ± standard deviation of six independent replicates for each sample.

#### 2.2.6 Bulk Wettability (Swelling Test)

To ascertain the bulk wettability percentage, both untreated and annealed samples were analyzed and compared with each other. Prior to testing, the samples were conditioned at 23 °C and 50% relative humidity. Three specimens of each sample were weighed and immersed in deionized water. For each measurement, the sample films were thoroughly dried using absorbent tissue and weighed. The immersion durations were 1, 18, 121, 257, and 334 h. The results are presented as averages of three replicate measurements. The swelling percentage in both scenarios was calculated using eq 3 as follows

1 $$\left(S\%\right)={(W}_{S}-W_{D})/W_{D}\times 100$$

where *W*_S_ and *W*_D_ are the swollen and dry weights of the films (PVAc and nanocomposites), respectively.

#### 2.2.7 Water Vapor Transmission Rate

The water vapor transmission rate (WVTR) was assessed according to ASTM E96/E96–23, employing a desiccant method. Circular specimens were prepared via laser cutting and affixed to the upper edges of Petri dishes using a sealing material (butyl sealing tape, ST150). Subsequently, the sealed dishes were placed in a controlled-humidity chamber (BINDER GmbH, KBF-S 240) maintained at 38 °C and 80% relative humidity (RH). The moisture uptake by the desiccant was monitored by periodically weighing the dishes throughout the test duration. For each sample group, a blank specimen (without a desiccant) was used to account for the water adsorption by the film.

The water vapor transmission rate was determined as the mean of three replicate measurements and expressed in g h^–1^ m^–2^. This rate was calculated from the slope of the mass change over time (Δ*G*/Δ*t*), as specified in eq 4.

2 $$WVTR=\frac{G}{t\times A}=\frac{\left(G/t\right)}{A}$$

Here, *G* denotes the steady-state weight gain (g), *t* represents the elapsed time (h) corresponding to *G*, and *A* signifies the exposed dish mouth area (m^2^). The steady-state conditions were determined by plotting the weight change against time, with the data corrected using a blank specimen. The coefficient of determination (*R* ^2^) was used to evaluate the linear correlation. Subsequently, the permeance was calculated using eq 5.

3 $$Permeance=\frac{WVTR}{\Delta p}=\frac{WVTR}{S\cdot \left(R-R\right)}$$

Here, Δ*p* represents the difference in vapor pressure, and *S* signifies the saturation vapor pressure at the specified test temperature. The relative humidity inside the dish was assumed to be 0% because of the presence of a desiccant. The permeability was determined by multiplying the calculated permeance by the thickness of each film, as described in eq 6.

4 Permeability=permeance×Thickness

#### 2.2.8 Dynamic Mechanical Analysis (DMA)

The viscoelastic properties of the samples were examined using a DMA Q-series dynamic mechanical thermal analyzer (TA Instruments, New Castle, DE, USA) in the film tension mode. The glass transition temperature (*T*_g_) of the rectangular samples was determined under the following conditions.^26^ The samples were subjected to a temperature range from −20 to 110 °C at a heating rate of 3 °C min^–1^, with a frequency of 1 Hz and a strain amplitude of 5% in an air environment. The storage modulus (*E*′), loss modulus (*E*″), and tan delta were recorded as functions of temperature.

Short-term creep tests were conducted in the tension mode at a constant temperature. All specimens were equilibrated at 25 °C for 5 min prior to the initiation of the creep test. During the short-term creep tests at 25 °C, a stress of 1 MPa was applied and maintained for 10 min, followed by a recovery period of 40 min. The creep percentage quantifies the total strain observed at a given time and is expressed as a percentage. Creep compliance, a normalized parameter that remains independent of the applied stress, facilitates the comparison of the intrinsic viscoelastic properties of materials. This was determined by calculating the ratio of the strain to the applied stress.

#### 2.2.9 Tensile Test

Tensile tests were conducted in accordance with the EN ISO 527–1:2019 standard (specimen type 2; EN ISO 527–3) utilizing a Tinius Olsen H10KT testing machine (Horsham, PA, USA) equipped with Horizon software (version 10.3.1.10) and a 100 N load cell (type: FBB-100N). For each formulation, a minimum of six dumbbell-shaped specimens were prepared using a laser-cutting machine and tested at two grip speeds of 1 and 5 mm/min. Prior to testing, all samples were conditioned in a climate chamber at 23 °C and 50% relative humidity (RH) for 48 h. The mean values and standard deviations were calculated and reported. The conditions in the tensile testing room were maintained at 22 °C and 40% RH.

#### 2.2.10 Thermogravimetric Analysis

Thermogravimetric analysis (TGA) was performed using a Q500 instrument (TA Instruments, New Castle, DE, USA). The samples underwent thermal treatment, during which the temperature was elevated from 35 to 650 °C at a rate of 5 °C per minute under a continuous nitrogen flow of 60 mL per minute. Mass losses corresponding to 2, 5, and 50 wt % were recorded at these temperatures. The onset temperatures of the first and second major degradation steps and the residual mass at 650 °C were determined from the TGA curves. Thermogravimetric (TG) and derivative thermogravimetric (DTG) curves were obtained using the instrument and analyzed using the TA Universal Analysis 2000 software.

#### 2.2.11 Statistical Analysis

Statistical analyses were conducted using Minitab Statistical Software (Minitab LLC, version 22, State College, PA, USA). A general linear model (GLM) and one-way analysis of variance (ANOVA) were used to assess the impact of the experimental factors on the measured responses. One-way ANOVA was applied when a single factor was considered, facilitating the comparison of the mean values across groups. Prior to model fitting, homogeneity of variances was evaluated using Levene’s test. In instances where equal variance was not present, the Box-Cox transformation was applied to the response variables. Statistical significance was set at a 95% confidence level (α = 0.05). After identifying significant effects, multiple comparisons were conducted using Tukey’s posthoc test to ascertain statistically distinct groups. Potential outliers were assessed in accordance with ASTM E178–21, and data points were excluded only when justified by both the statistical evidence and identifiable experimental causes.

## 3 Results and Discussion

### 3.1 Nanocomposite Latex

The nanocomposite latex was successfully synthesized via the procedure described in Section 2.1.1, and the latex remained stable for at least six months after the synthesis. The total solid content (TSC) was determined by weighing triplicate samples before and after drying at 105 °C, and the average values were reported. All polymerizations were performed at a TSC of approximately 49–51 wt %. All nanocomposites exhibited shear-thinning behavior, as described in a previous study.^3^ The total solid content (TSC) was assessed in accordance with ASTM D4209–07(2019), and the CNCs content was reported on a wet and dry basis. The findings are presented in Table 2.

**Table 2:** Theoretical TSC, the Measured TSC, and the Calculated CNCs Content for the Samples in Both Wet and Dry Conditions

Sample formulation	TSC (%) Theoretical	TSC (%) (measured)	CNCs content in latex (wet basis, %)	CNCs content (dry basis, %)
PVAc	49.4	49.0	0	0
PC3	48.9	48.0	0.31	0.64
PC6	49.6	48.9	0.61	1.26
PC9	50.0	49.9	0.92	1.89
PC12	48.9	49.0	1.23	2.51

### 3.2 Scanning Electron Microscopy

The SEM micrographs (Figure 1) illustrate the morphologies of the PVAc and nanocomposite freeze-dried particles. The pristine polyvinyl acetate particles were relatively small, measuring significantly less than 1 μm. The results showed that the particle size increased with increasing CNCs concentration. This phenomenon was accompanied by variations in the particle size and shape, as observed in Figures D and E, where the CNCs concentration reached its maximum value. This effect has previously been documented by Bayat, Meek,^27^ who reported an increase in particle size attributable to the addition of sulfated CNCs, along with a more dispersed particle size distribution.

**Figure 1:**
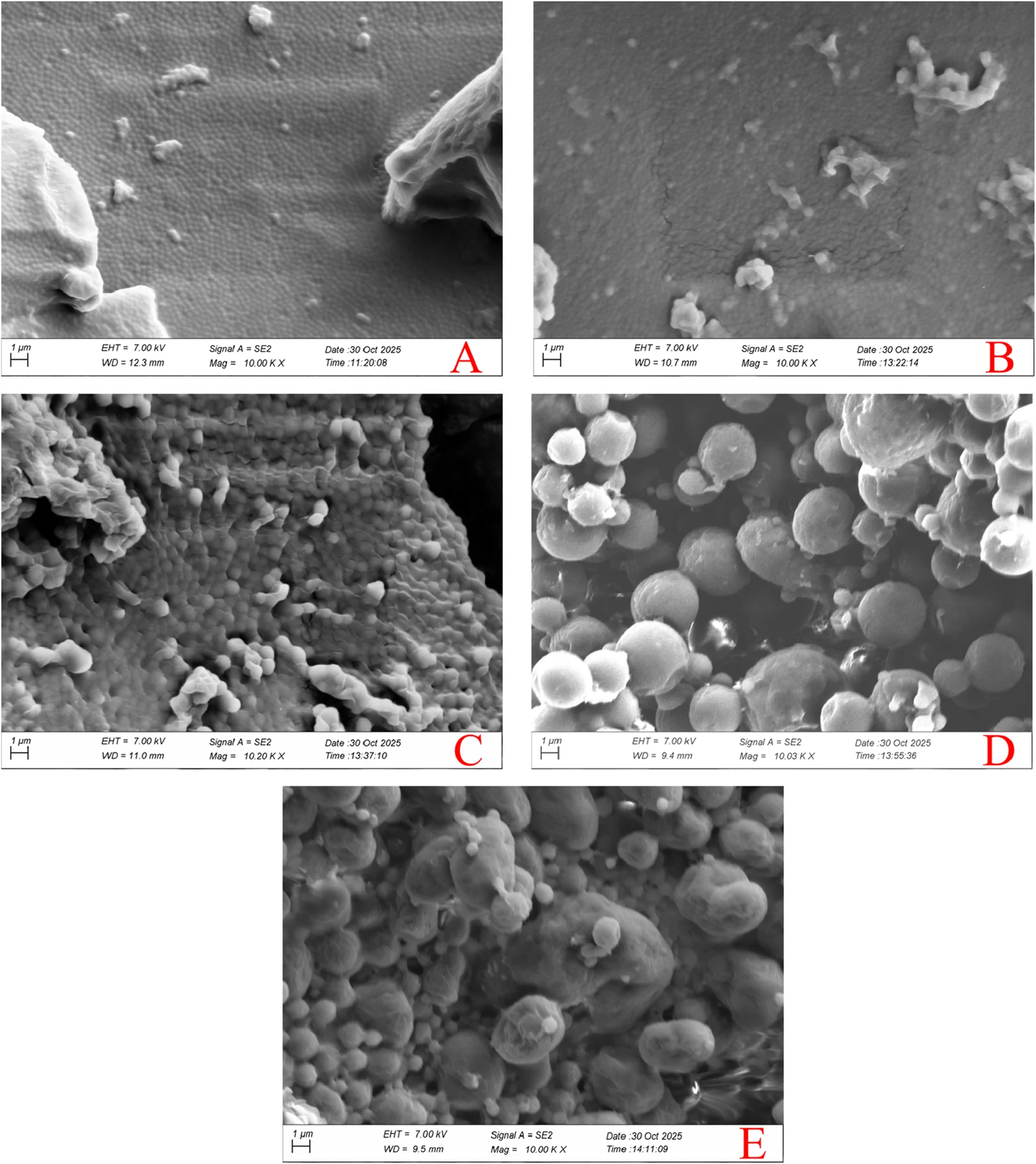
SEM micrographs of PVAc and nanocomposite particles: (A) PVAc, (B) PC3, (C) PC6, (D) PC9, and (E) PC12.

Another study by Mabrouk, Dufresne^3^ reported that the surface contours of particles in nanocomposites deviated from a spherical configuration owing to MPEG, with smaller particles exhibiting a tendency to cluster together. They attributed these irregular surfaces to CNCs bound around the particles. Our previous study indicated that in the absence of MPEG, the polymer particles exhibited an increase in size with the augmentation of CNCs concentration while maintaining their spherical shape.^13^ A comparison of the SEM micrographs of PC12 with the SEM in our previous study^13^ shows that a higher concentration of CNCs in the presence of MPEG resulted in the formation of larger, irregularly shaped particles. According to the findings of Mabrouk, Dufresne,^3^ in the absence of MPEG, CNCs tend to remain dispersed within the aqueous phase rather than adsorbing onto the surfaces of particles. It is noteworthy that the application of CNCs in its dried form may plausibly account for the incomplete dispersion of CNCs in water, which could lead to potential aggregation.^10^

### 3.3 ^13^C MAS NMR Spectroscopy

The NMR spectra of CNCs, PVAc, and the two nanocomposites (PC3 and PC12) elucidate the structural role of CNCs within the polymer matrix (Figure 2). The CNCs spectrum exhibits as expected the characteristic signals of cellulose, with peaks corresponding to C1 (∼105 ppm), C4 (peaks split into two regions of crystalline (∼89 ppm) and amorphous (∼84 ppm)), and C2, C3, C5 (∼72–76 ppm), in addition to C6 (∼65 ppm).^28^ The spectrum of polyvinyl acetate (PVAc) is characterized by prominent signals associated with the polymer backbone and side groups. These include the carbonyl (C=O) peak at 170.4 ppm, as well as methine (CHO) at 67.2 ppm, methylene (CH_2_) at 40.5 ppm, and methyl (CH_3_) groups at 21.2 ppm. The spectra of PC3 and PC12 were magnified by a factor of 20, and the peaks corresponding to the C1 and C4 carbons of CNC were clearly observed. The magnitude of the CNC peaks is smaller than the spinning sidebands noted in Figure 2 as ssb, which occur at multiples of the sample spinning frequencies (13.5 kHz). By analyzing the normalized spectra of CNC and PVAc, we determined that the peaks associated with PVAc remained predominant, suggesting that PVAc governed the overall structural characteristics of all samples.

**Figure 2:**
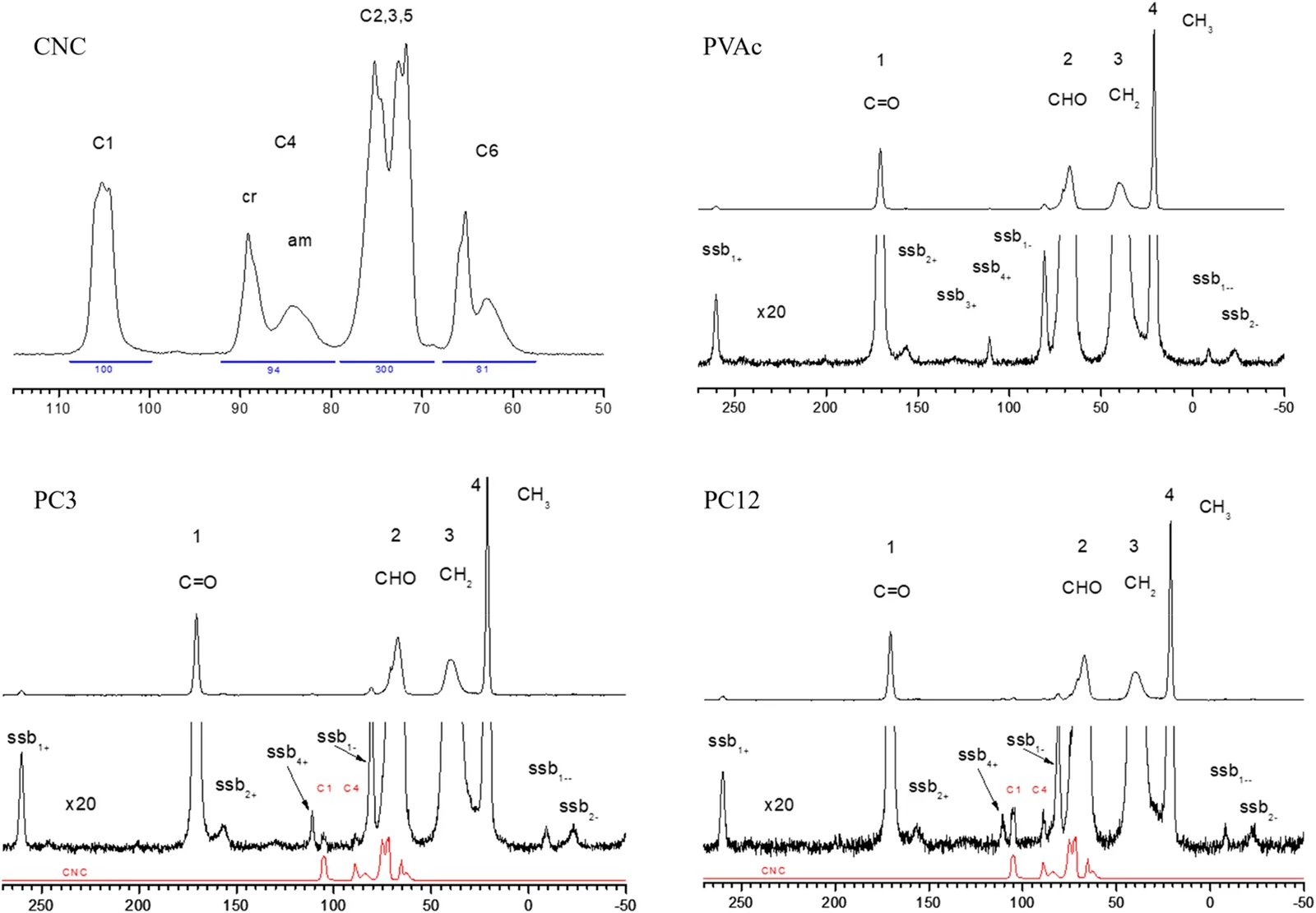
Solid-state ^13^C NMR spectra of CNCs, PVAc, and nanocomposites (PC3 and PC12).

The absence of significant changes in the chemical shifts of the samples suggests that the interaction is predominantly physical, such as through hydrogen bonding or interfacial association, rather than involving the formation of new covalent bonds. The NMR spectrum confirms that all polymeric species are present in the sample, including both the PVAc polymer and any PVAc that may be attached to the CNCs surface.

### 3.4 Polarization Optical Microscopy

All films were prepared on glass slides with a wet thickness of 200 μm and subsequently dried in a convection oven at 60 °C. The PVAc film without CNC exhibited a completely flat surface devoid of discernible polymer particles and was therefore excluded from the microscopy investigation. Figure 3 shows the nanocomposites with varying loadings and heat treatments. Although the drying process was conducted at temperatures surpassing the glass transition temperature, polarized images indicated that particle coalescence was limited, particularly at higher CNCs concentrations. The white arrows in Figure 3a,d show the presence of spherical particles, indicating restricted coalescence.

**Figure 3:**
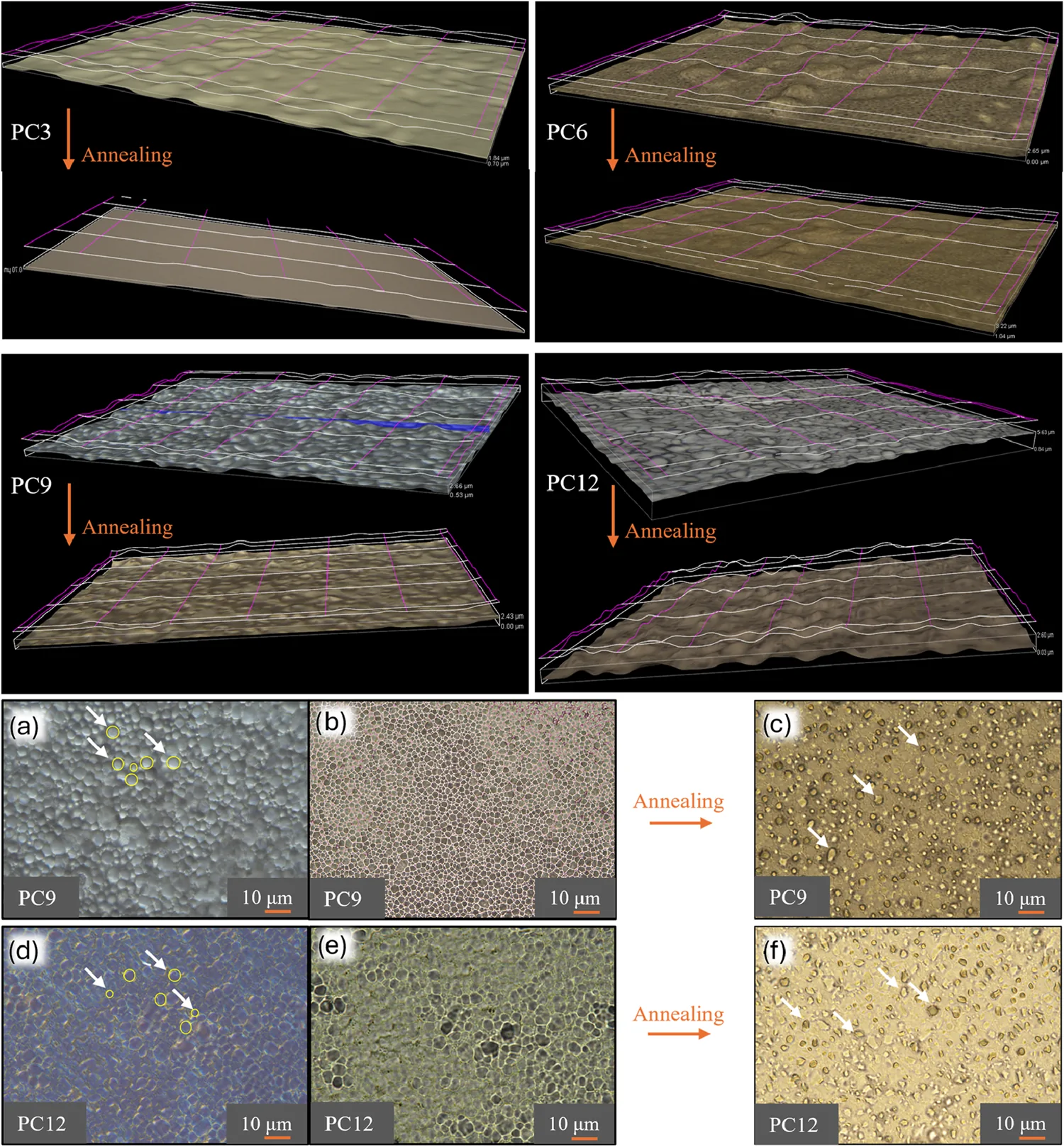
Top four pictures: Topographic images of the nanocomposite samples PC3, PC6, PC9, and PC12 before and after annealing (100× reflected light, bright field). Below pictures identified as (a)–(f): (a, b) optical image of PC9 showing the same area acquired under two imaging conditions after drying at 60 °C: (a) transmitted light, dark field, (b) reflected light, bright field, (c) microscopic image of PC9 after annealing (reflected light, bright field), (d, e) optical image of PC12 showing the same area acquired under two imaging conditions after drying at 60 °C: (d) transmitted light, dark field, (e) reflected light, bright field, (f) microscopic image of PC12 after annealing (reflected light, bright field).

Polarized microscopy images provide insights into the evolution of the surface morphology and particle organization of the nanocomposites as a function of the CNCs content and post-treatment. As illustrated in Figure 3, the incorporation of CNCs increased surface heterogeneity. With increasing CNCs concentration (from PC3 to PC12), the films exhibited a more pronounced uneven topography and the emergence of a mosaic-like pattern (Figure 3b,3e). Nevertheless, the dispersibility of the CNCs cannot be solely assessed based on the roughness of the surface; it is essential to consider the interaction between the CNCs and the PVAc matrix.^29^ The study conducted by Malmir, Montero^30^ revealed that an increase in CNCs content resulted in enhanced surface roughness and irregularity. This behavior can be attributed to multiple factors, such as hydrogen bonding between the polymer chains and CNCs, electrostatic repulsion, and potential CNC agglomeration. In addition, the presence of surfactants, MPEG, residual radicals, and CNCs within the polymer matrix may hinder polymer chain interdiffusion and promote the formation of discrete domains.^29,31^

Postannealing analysis revealed no significant reduction in macroscopic surface irregularities, as shown in the topographical images (Figure 3). Nevertheless, a significant transformation was evident in the coalescence of the particles by the disappearance of the mosaic pattern, indicating enhanced particle interdiffusion and coalescence. These observations indicate that annealing facilitates structural rearrangement within the film, even though it does not eliminate surface irregularities, which has been marked with white arrows in Figure 3c,f.

These findings suggest that while annealing enhances particle fusion, the presence and concentration of CNCs influence film morphology and coalescence.

### 3.5 Transmittance Measurements

In CNCs nanocomposites, variations in transmittance can to some extent indicate the dispersion quality within the matrix.^29^ The transmittance results demonstrated a decrease in optical transparency as the CNCs content increased, with a more significant effect observed at higher concentrations (Figure 4). A significant decrease in transmittance typically indicates particle aggregation, wherein clusters become sufficiently large to cause notable light scattering.^25^

**Figure 4:**
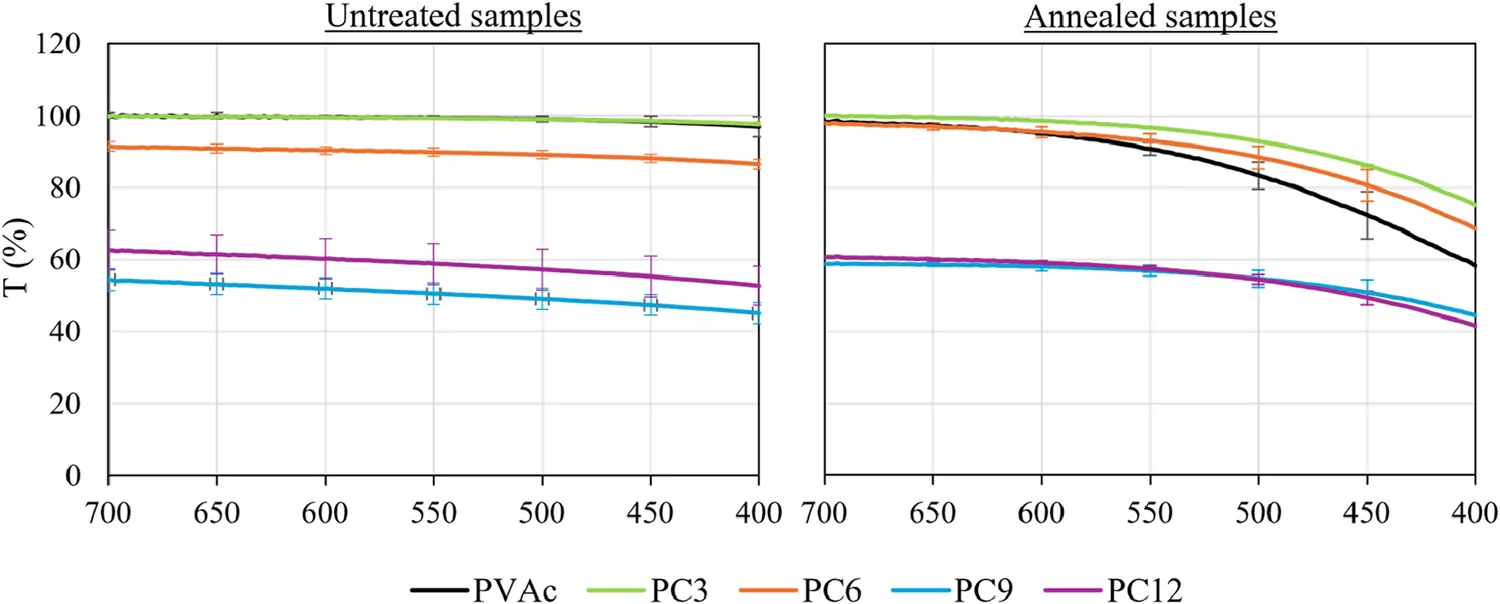
Spectrometric graphs: (left) untreated samples, (right) annealed samples.

For untreated PVAc films, the high transmittance across the measured wavelength range suggests a homogeneous and amorphous polymer matrix with minimal light scattering. Incorporation of CNCs in PC3 sample caused negligible changes in transmittance compared to PVAc, suggesting good dispersion without noticeable agglomeration that could scatter visible light.^32^ As the CNCs concentration increased, a more significant decline in transmittance was observed, as previously reported by Mabrouk, Dufresne.^3^ This phenomenon may result from the simultaneous occurrence of CNCs aggregation, formation of CNCs-rich domains, and surface irregularities, which collectively enhance the effective scattering centers and lead to increased turbidity. Scanning electron microscopy (Figure 1) indicated that an increase in the cellulose nanocrystals within the samples led to larger particle sizes, which could have contributed to the observed reduction in transmittance. Polarization microscopy observations further substantiate this interpretation by revealing mosaic-pattern features (Figure 3b,e) and increased surface irregularity (Figure 5) at elevated CNCs loadings.

**Figure 5:**
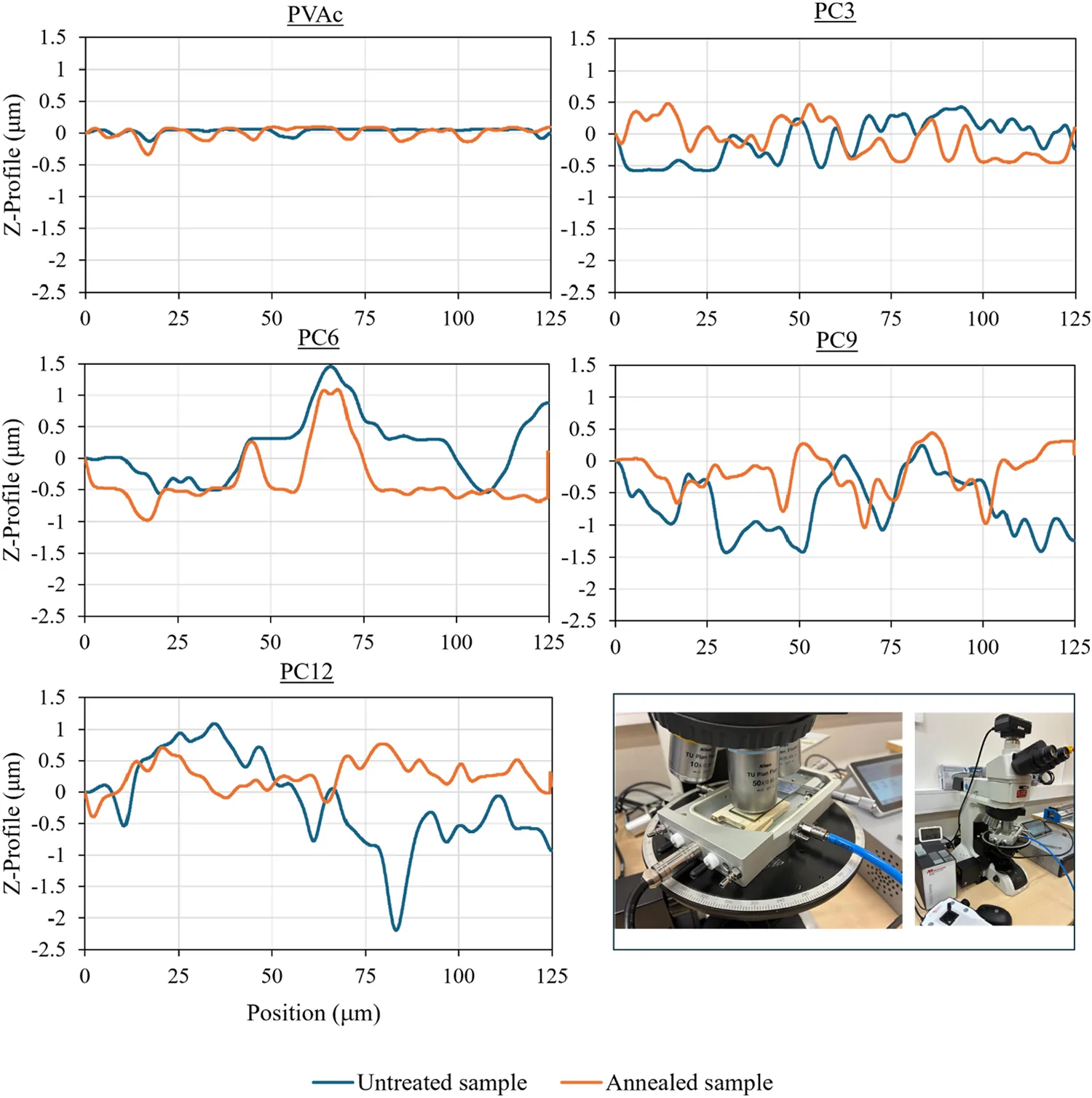
Z-profile of untreated and annealed films for PVAc, PC3, PC6, PC9, and PC12 is presented. The lower photograph on the right illustrates the Linkam apparatus used for annealing the films prepared on glass slides.

For clarity, Figure 5 presents the local z-profiles of the samples, comparing the irregularities before and after annealing. The results indicate that annealing alters the surface topography. For the PVAc film, annealing amplified the surface irregularities. Although the extent of surface unevenness was not significant, the annealed surface displayed greater roughness than the untreated film surface. In contrast, the annealing effect on PC3 was not evident; however, nanocomposites with higher cellulose nanocrystal contents, specifically PC6 and PC9, exhibited smoother surfaces with fewer surface irregularities. The most notable effect was observed in PC12, where the film exhibited a smoother surface after annealing.

As shown in Figure 4, both untreated PVAc and PC3 exhibited identical transmittance values across the entire wavelength spectrum. After annealing, a reduction in transmittance was observed, particularly at lower wavelengths. A comparison between PVAc and the nanocomposite with the lowest CNCs loading (PC3) revealed a more pronounced decrease in transmittance for PVAc. The influence of CNCs on transmittance was more distinctly observable in PC6, which contained a moderate amount of CNCs. While untreated PC6 initially displayed lower transmittance than PVAc, annealing resulted in an increase in the transmittance of PC6, aligning it closely with the values observed for PVAc postannealing. This phenomenon may be attributed to the combined effects of particle coalescence and surface smoothness induced by annealing. Finally, the negligible effect of annealing on PC9 and PC12 may be attributed to their larger particle size and surface irregularities

### 3.6 Water Contact Angle

The water contact angle reported here should be considered an apparent contact angle, because the measured value is influenced by the surface chemistry, roughness, heterogeneity, and possible water penetration into the film. By examining how drop volume and contact angle evolve over time, it is possible to determine parameters like surface roughness, and bulk penetration.^33^ Nonetheless, this study did not report variations in droplet volume.

Figure 6 presents the water contact angle measurements of the PVAc and nanocomposite films conducted under laboratory conditions, both prior to and after annealing. The untreated samples exhibited a comparatively lower contact angle, indicating hydrophilicity. After annealing, PVAc exhibited a slight increase in contact angle and a reduced variation between 0 and 10 s, suggesting that the thermal treatment diminished the propensity of water to rapidly penetrate or spread across the surface.

**Figure 6:**
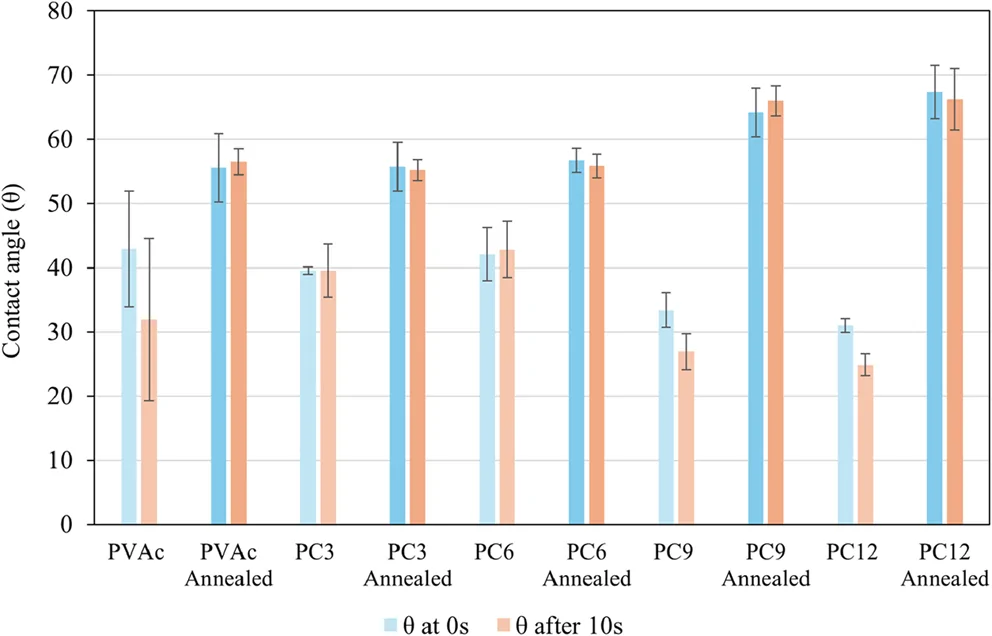
Water contact angles of PVAc and nanocomposite films before and after annealing, measured immediately after droplet deposition (0 s) and after 10 s under laboratory conditions. Error bars represent standard deviations.

For the nanocomposite samples, annealing resulted in a clear increase in the contact angle. This behavior may be attributed to improved film consolidation after annealing, including enhanced latex particle coalescence, chain packing, and interfacial connections between the polar groups of PVAc and CNCs.

The contact angle was elucidated by comparing the results of the untreated samples. There was almost no clear difference in the water contact angles of the untreated samples. For the nanocomposites, the SEM images of the freeze-dried samples (Figure 1) showed that higher CNCs loading led to larger particles. The absence of coalescence and interdiffusion, as discussed in Section 3.3, promoted the formation of nanocomposite film with larger particles which may facilitate water diffusion. Moreover, the inherent polarity of sulfated CNCs from either available hydroxyl groups or sulfate half-ester groups increases the polarity and hydrophilicity of the surface of nanocomposite film.^34^ Consequently, the untreated PC9 and PC12 samples demonstrated lower contact angles than the samples with lower CNCs concentrations. For nanocomposites, the interaction between the CNCs and PVAc is anticipated to occur primarily via hydrogen bonding between the hydroxyl groups of CNCs and the ester carbonyl groups of PVAc.^34,35^ For untreated samples, it is plausible that the majority of the particles remained unconnected.

Annealing may affect both the polymer and the interaction between the polymer and cellulose nanocrystals. Research conducted by Gonzalez and Alvarez^36^ demonstrated that poly­(vinyl alcohol) hydrogels exhibit enhanced structural integrity and decreased water absorption and swelling properties after annealing. However, these benefits are associated with a decrease in transparency.^36^ Our study corroborates these findings, demonstrating that PVAc exhibits reduced water absorption (Figure 7) and diminished transparency after annealing (Figure 4).

**Figure 7:**
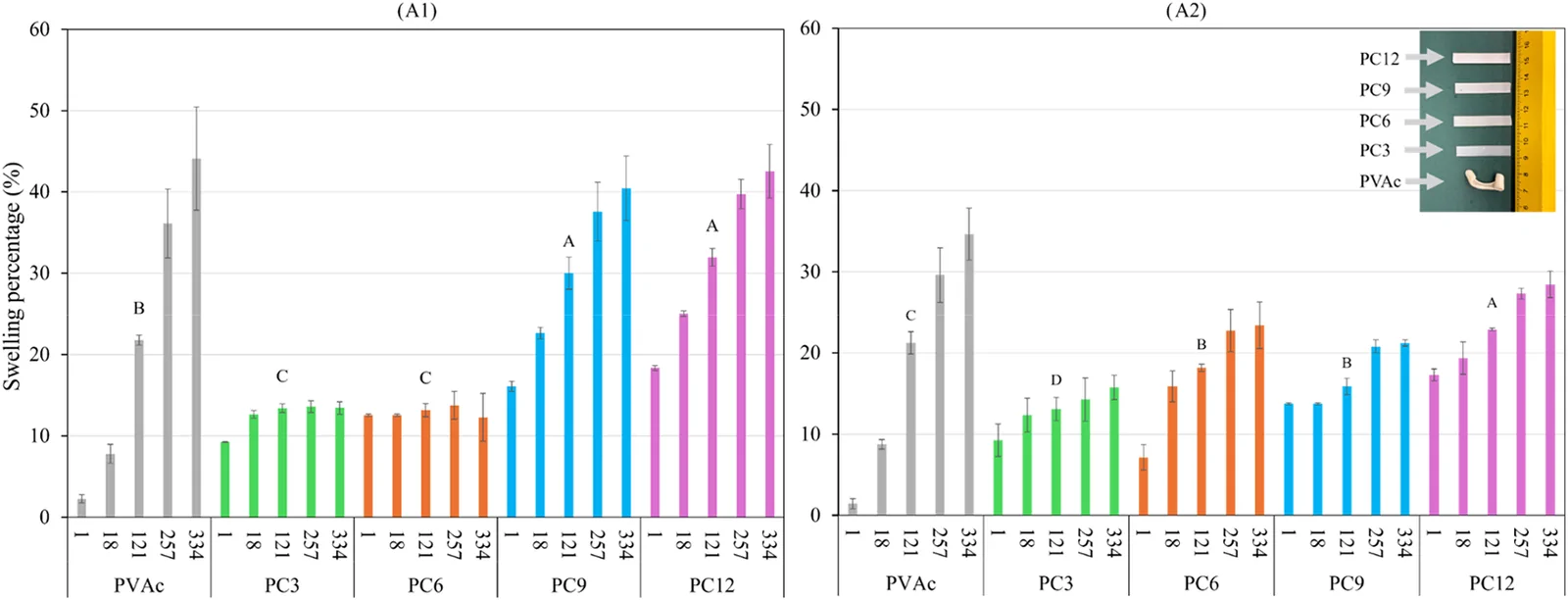
Swelling behavior of PVAc and nanocomposite samples as a function of immersion time. (A1) Untreated samples and (A2) annealed samples. The swelling percentages were measured after 1, 18, 121, 257, and 334 h of immersion in water. The bars represent the mean values, with error bars indicating the standard deviation. Different letters above each group denote statistically significant differences among the groups. The inset (A2) shows the appearance of the samples after swelling testing.

Structural modifications can reduce the accessibility of polar groups and hinder the rapid penetration of water into the film by making the material more compact and structurally robust. Polarized microscopy examination (Figure 3) revealed that the structural pattern of the film surface exhibited particle coalescence and the disappearance of the mosaic pattern after annealing. This leads to fewer openings for water penetration and decreases the flexibility of the network to expand during swelling.^36^ Although CNCs contains abundant polar groups, the annealing process may decrease the exposure of accessible polar groups by development of stronger hydrogen bonding networks between PVAc and CNCs.^37^ The contact angles of the untreated and annealed nanocomposites revealed a distinct increase in the contact angle, with negligible variation between θ at 0 and 10 s at higher CNCs loadings. The highest contact angle observed for annealed PC9 and PC12 can be attributed to the combined effects of surface roughness and a reduction in the number of available polar or hydroxyl groups. The resulting uneven surface inhibited the deposited water from readily contacting or wetting the substrate surface.

The findings suggest that annealing enhances the apparent water contact angle of the PVAc/CNCs nanocomposite films, owing to the synergistic effect of film coalescence and reduction in availability of polar groups.

### 3.7 Bulk Wettability (Swelling Test)

The group bar charts in Figure 7 show the swelling percentages of the untreated and annealed samples. All nanocomposite films remained structurally stable after the swelling tests, whereas the PVAc film did not retain its structural integrity (Figure 7A2, insert). The swelling data were fitted using a general linear model, and differences among samples were evaluated using analysis of variance (ANOVA), followed by multiple comparison analyses.

For the untreated samples, PC3 and PC6 exhibited the lowest mean swelling (∼12%), with no statistical difference between them. PVAc showed an average swelling value (18.8%), whereas PC9 and PC12 presented the highest swelling (∼30%) and were not significantly different from each other. At the initial immersion stage (1 h), PVAc exhibited the lowest water uptake, which differed from that of the other samples. During the experiment, the swelling of untreated PC3 and PC6 remained relatively constant at approximately 12–13%, whereas the other formulations exhibited a significant increase in water absorption over time as a function of immersion duration.

The swelling behavior of the annealed samples showed that PC3 exhibited the lowest mean swelling (∼12.7%), which was significantly lower than that of the other nanocomposites. The annealed PVAc exhibited a relatively low swelling mean (∼15.9%). PC6 and PC9 displayed similar swelling behaviors with no statistical difference between them (∼16.8%), whereas PC12 showed the highest mean swelling and differed from the other formulations (∼22.8%).

At the early stage (1 h), the annealed PVAc showed the lowest water uptake (∼1%), followed by PC3 and PC6, with approximately 9 and 7%, respectively.

The effect of annealing on swelling indicated that, except for PC3, all the samples exhibited a statistically significant change. After annealing, the mean swelling of PC6 increased by approximately 31%, whereas reductions were observed for PC9 (∼68%), PC12 (∼35%), and PVAc (∼18%). Among these samples, PC9 exhibited the most pronounced response to annealing, with the largest reduction in swelling observed. The most significant reduction in swelling observed in PC9 and PC12 may be attributed to the coalescence of particles, which diminishes the available pathways between particles, as illustrated in Figure 3. The disappearance of the boundary lines for PC9 and PC12 following annealing, as illustrated in Figure 3 (transitioning the surface from b to c for PC9 and from e to f for PC12), may potentially obstruct the pathway for water diffusion into the film.

### 3.8 Water Vapor Transmission Rate

Figure 8 shows the cumulative weight gains as a function of time for untreated and annealed samples. The linear regions of the curves correspond to the steady-state water vapor transmission, where a steeper slope indicates a higher WVTR. Among the untreated films, PVAc exhibited the lowest steady-state mass-gain gradient and the greatest thickness (0.6 mm) (Figure 8A1, insert). This suggests that the relatively low WVTR is primarily influenced by thickness rather than the superior intrinsic barrier properties.

**Figure 8:**
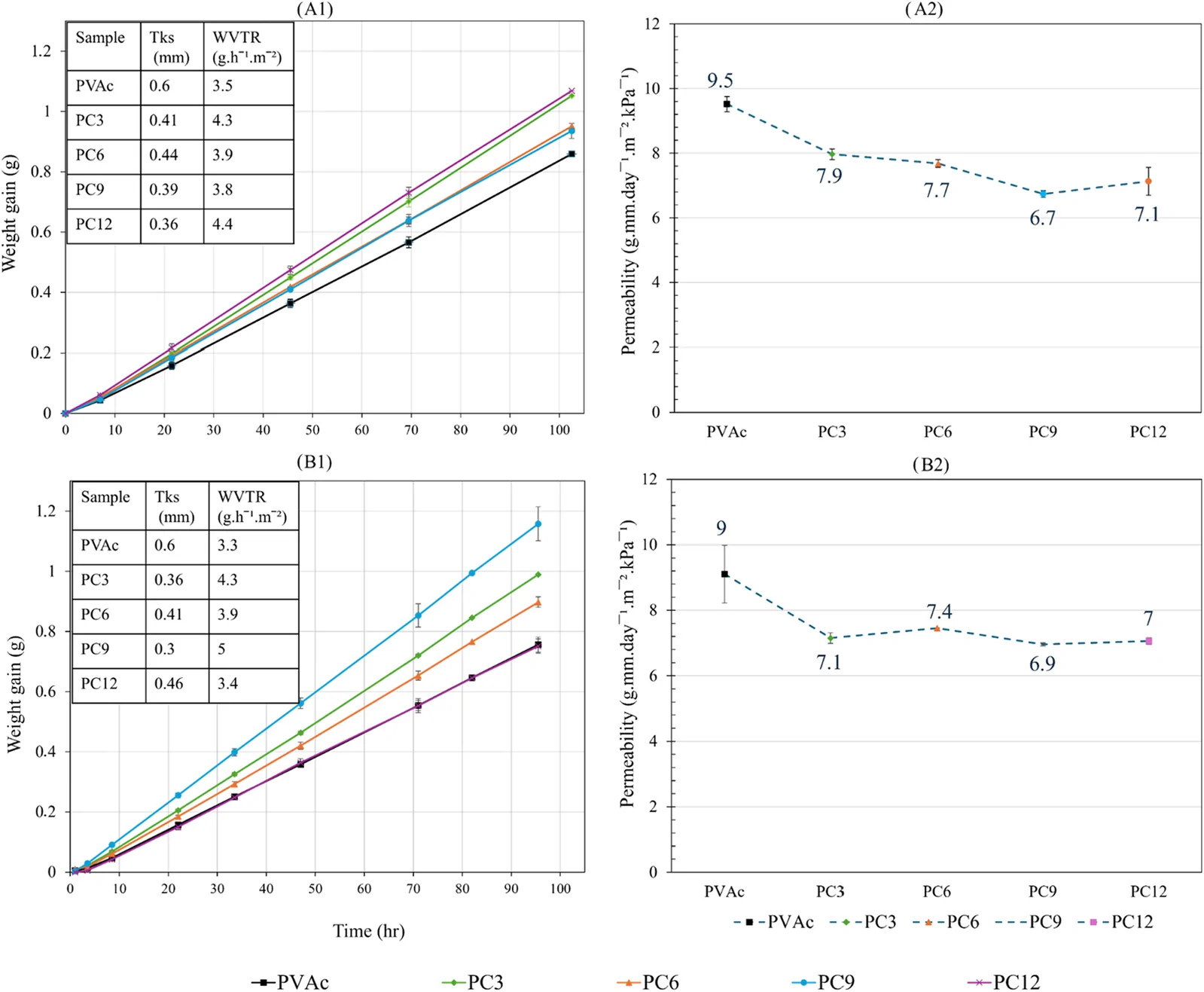
WVTR results for untreated and annealed films: (A1) weight gain of untreated films, (A2) permeability of untreated films, (B1) weight gain of annealed films, and (B2) permeability of annealed films.

This interpretation is corroborated by Figure 8A2, which demonstrates that PVAc exhibits the highest permeability among the untreated samples. In contrast, all the nanocomposite films exhibited lower permeability, indicating reduced intrinsic water vapor transport through the film. Among the untreated samples, PC9 demonstrated the lowest permeability, which was 29% lower than that of PVAc, and it did not differ significantly from that of PC12. In contrast, PC3 and PC6 showed similar permeability values, each approximately 20% lower than that of PVAc. The reduced permeability of the nanocomposites may be attributed to the increased tortuosity induced by the CNCs phase; this effect is expected to intensify with higher CNCs loading, consistent with the lower permeability observed for PC9 and PC12.

After annealing (Figure 8B1), the variations in the WVTR were attributed to the combined effects of changes in thickness and annealing-induced structural modifications.

The WVTR behavior of the annealed samples is shown in Figure 8B1. For PVAc, the film thickness was similar to that of the untreated sample, and the observed reduction in WVTR from 3.5 to 3.3 indicates a slight intrinsic decrease in permeability. For the annealed nanocomposite films, except for PC12, the annealed films had a lower thickness (Figure 8B1, insert).

According to the thickness effect, the WVTR of PC3 was anticipated to increase to approximately 4.9 (eqs 4–6). However, the experimentally determined WVTR was 4.3, indicating an intrinsic enhancement in the barrier performance that surpassed the thickness effect. A similar but modest behavior was observed for PC6, where a slight decrease in thickness led to a predicted WVTR of approximately 4.2, whereas the measured value was 3.9, indicating a moderate reduction in the permeability.

In contrast, the thickness of PC9 decreased from 0.39 mm to 0.3 mm. The agreement between the predicted and measured values (4.9 and 5, respectively) indicates that the increased WVTR was primarily governed by the thickness reduction, with almost no intrinsic improvement in the permeability. For PC12, the annealed film was thicker than the untreated film (0.46 and 0.36 mm, respectively), resulting in a reduced WVTR, which was largely attributed to the thickness effect, accompanied by only a minor intrinsic improvement in permeability.

Thickness normalization indicated that PVAc had the highest permeability among the untreated samples, and annealing did not significantly affect the permeability of PVAc. Upon annealing, the intrinsic barrier enhancement was most pronounced for PC3, where the WVTR decreased despite a reduction in film thickness, indicating a substantial decrease in permeability. This behavior may be attributed to annealing-induced structural rearrangements, including improved filler–matrix interactions and the reduction of micro voids, surface defects, and film nonuniformities.

### 3.9 Dynamic Mechanical Analysis

Recent studies have consistently reported an increase in the glass transition temperature (*T*_g_) with the addition of inorganic nanofillers to polymers. This could be attributed to the restricted polymer chain mobility owing to polymer–nanofiller interactions, interfacial adhesion, and network formation.^38^ Dynamic mechanical analysis (DMA) is a valuable tool for examining the chemical composition and physical molecular structure of polymers. The chemical composition is instrumental in determining the loci of changes in mechanical properties, whereas the physical molecular structure dictates the mechanisms underlying these changes.^26^ Moreover, DMA outcomes are subject to variations based on the processing conditions, product characteristics, and molecular structure. Previous research by Cangialosi, Boucher^38^ indicated that the heterogeneous structure of a nanocomposite can decelerate physical aging by imposing topological constraints (structural heterogeneity) on the polymer network. These constraints restrict the segmental dynamics of the chains.^39^ Additionally, the presence of a layer with reduced mobility at the interface between the polymer and nanoparticles, or strong interfacial interactions such as hydrogen bonding, may also contribute to this effect.^38^

The impact of CNCs incorporation on the material response following various formulations and thermal treatments was assessed for four different groups: untreated samples (group 1), heat treated samples at 500 and 1000 h (groups 2 and 3, respectively), and annealed samples (group 4). A general linear model (GLM) was applied to evaluate the effects of sample formulation, treatment, and the interaction between formulation and treatment on the glass transition temperature and storage modulus. As the free volume continues to expand with increasing temperature, glass transition occurs, allowing large chain segments to move. *T*_g_ was measured using three distinct methods: the point at which the storage modulus *E*′ began to decline, the peak of the loss modulus *E*″ curve, and the peak of the tan δ curve. The variation between different measurements can be as large as 22 °C.

In the untreated samples, the height of the tan δ peak decreased with increasing CNCs content (Figure 9a1). A higher tan δ peak signifies increased energy dissipation and internal friction, while a narrower peak indicates enhanced homogeneity within the material.^26,38,40^ Although heat treatment at 500 and 1000 h did not influence the height of the tan δ peak for the nanocomposite samples, it led to an increase in the height of the PVA peak, as depicted in Figure 9a2,a3. The annealing process had a notably greater impact on PVAc, PC3, and PC6, as evidenced by the increase in the height and decrease in the width of the tan δ peak (Figure 9a4). The effect of heat treatment was reduced in samples with high CNCs concentrations (PC9 and PC12; Figure 9a). This observation aligns with previous research on the influence of topological constraints on the segmental dynamics of chains.^39,41^

**Figure 9:**
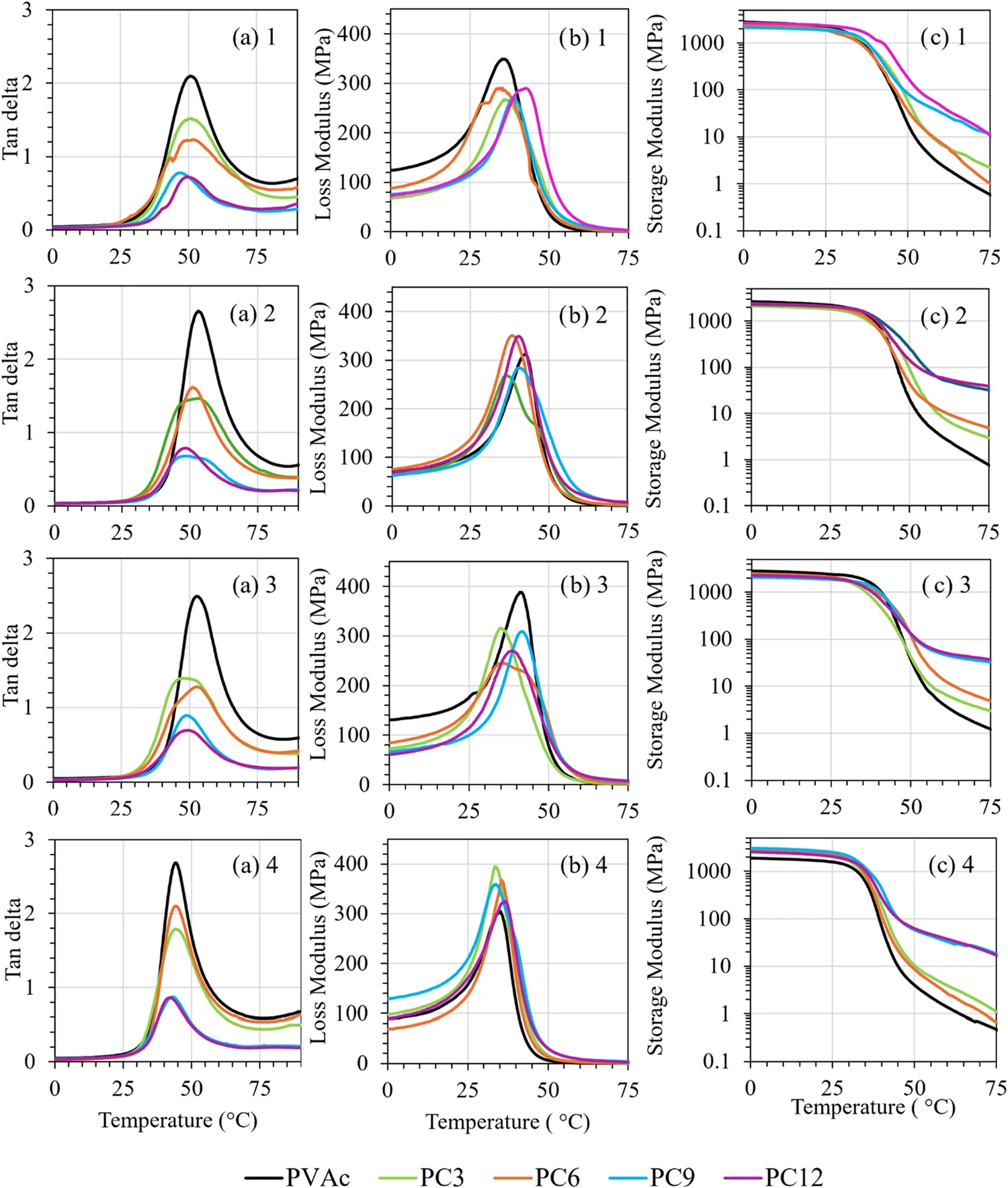
(a) Tan δ, (b) loss modulus (*E*″), and (c) storage modulus (*E*′) of PVAc and nanocomposites: (1) untreated sample, (2) samples after 500 h of heat treatment, (3) samples after 1000 h of heat treatment, and (4) annealed samples.

Several studies have shown that *T*_g_ may remain unchanged or even decrease, depending on factors such as nanoparticle dispersion, polymer–filler interactions, and the extent of free volume introduced into the matrix.^23^ Khan, Qi^42^ demonstrated that when the interaction strength between polymers and nanoparticles exceeds a critical threshold, *T*_g_ becomes largely independent of nanoparticle loading. Conversely, below this critical interaction strength, the more pronounced depression of *T*_g_ at higher nanoparticle concentrations is attributed to the diminished adsorption of nanoparticles on the polymer segments and the initiation of partial phase separation. In another study, Serenko, Roldughin^43^ reported that the depression of the glass transition temperature became more pronounced when polymer chains exhibited a weak affinity for the nanoparticle surface, particularly at high nanoparticle concentrations. These conditions led to insufficient wetting or coverage of the filler by polymer chains, resulting in phase separation. Consequently, the interfacial region was unable to immobilize the chains and instead exhibited increased mobility, partially functioning as a plasticizing phase. An increase in nanoparticle content results in a greater proportion of polymer chains being exposed to the mobile interfacial environment, thereby leading to a reduction in the overall glass transition temperature.

In our study, the samples exhibited negligible significant differences in *T*_g_ within each group. However, heat treatment and annealing induced variations between the groups (Table 3).

**Table 3:** Glass Transition Temperatures of the Sample Films Were Determined from Three Different Points: The Onset of the Storage Modulus Decline, the Peak of the Loss Modulus, and the tan δ Peak^a^,^b^

Sample	*T*_g_ at onset of *E*′ (°C)	*T*_g_ at *E*″ peak (°C)	*T*_g_ at tan δ peak (°C)	*E*′ at –20 °C (MPa)	*E*′ at 23 °C (MPa)
Untreated samples (group 1)					
PVAc	27.7 ± 3.0^A^	35.9 ± 2.3^A,ab^	49.7 ± 2.9^A,ab^	3106.2 ± 292^A,a^	2392,2 ± 79.5^A,a^
PC3	30.5 ± 4.8^A^	38.6 ± 4.2^A,ab^	49.8 ± 4.0^A,ab^	2309.5 ± 83.0^B,b^	1951.2 ± 110.5^C^
PC6	25.9 ± 5.9^A^	34.2 ± 5.3^A,ab^	48.5 ± 3.3^A,ab^	2589.2 ± 319^B,b^	1980.7 ± 71.3^C^
PC9	28.5 ± 5.6^A^	39.0 ± 2.3^A,ab^	47.1 ± 2.1^A,ab^	2409.7 ± 81.0^B,ab^	2053.2 ± 91.9^BC^
PC12	30.5 ± 2.7^A^	42.4 ± 1.4^A,ab^	49.8 ± 0.9^A,ab^	2583.6 ± 109^B,b^	2254 ± 113.5^AB^
Samples after 500 h heat treatment (group 2)					
PVAc-500h	32.6 ± 2^A^	41.9 ± 0.8^A,a^	53.0 ± 1.5^A,a^	2754.9 ± 635.3^A^	2265.4 ± 367.5^A^
PC3–500h	31.5 ± 3.6^A^	36.4 ± 4.3^A,a^	52.9 ± 4.4^A,ab^	2198.5 ± 334.7^A^	1861.9 ± 246.5^A^
PC6–500h	30 ± 3.2^A^	38.3 ± 2.7^A,a^	51.0 ± 2.5^A,ab^	2437.4 ± 230.4^A^	2026.5 ± 103.7^A^
PC9–500h	33.9 ± 2.6^A^	42.2 ± 3.3^A,a^	50.2 ± 4.0^A,ab^	2409.9 ± 412.0^A^	2113.7 ± 324.5^A^
PC12–500h	30.7 ± 1.7^A^	40.4 ± 3.3^A,a^	48.4 ± 3.4^A,ab^	2470.6 ± 335.4^A^	2102.2 ± 288.4^A^
Samples after 1000 h heat treatment (group 3)					
PVAc-1000h	31.7 ± 2.5^A^	40.5 ± 3.1^A,a^	52.9 ± 1.6^A,a^	2942.8 ± 321.2^A,a^	2461.8 ± 237.0^A^
PC3–1000h	29.7 ± 3.0^A^	37.1 ± 3.4^A,a^	48.3 ± 5.2^A,ab^	2413.5 ± 244.2^AB^	2004.9 ± 267.4^A^
PC6–1000h	30.4 ± 1^A^	40.0 ± 4.5^A,a^	50.3 ± 2.8^A,ab^	2567.0 ± 258.8^AB^	2129.5 ± 119.7^A^
PC9–1000h	32.7 ± 1.4^A^	42.4 ± 2.7^A,a^	49.0 ± 2.7^A,ab^	2177.5 ± 285.6^B^	1906.8 ± 288.3^A^
PC12–1000h	31.2 ± 1.7^A^	38.5 ± 1.9^A,a^	48.8 ± 1.5^A,ab^	2395.5 ± 234.3^AB^	2015.9 ± 201.3^A^
Annealed samples (group 4)					
PVAc(Annealed)	28.2 ± 2.1^A^	34.2 ± 1.4^A,b^	44.0 ± 0.5^A,ad^	1986.0 ± 88.8^B,c^	1619.2 ± 111.5^B,d^
PC3 (Annealed)	28.8 ± 1.2^A^	35.2 ± 2.4^A,b^	43.5 ± 2.4^A,bd^	2709.9 ± 310.6^AB,b^	2162.6 ± 222.6^AB,ad^
PC6 (Annealed)	29.5 ± 0.5^A^	35.6 ± 0.5^A,b^	44.0 ± 0.8^A,bd^	2870.9 ± 554.4^AB,b^	2306.8 ± 422.4^AB,ad^
PC9 (Annealed)	29.2 ± 3.6^A^	35.4 ± 3.3^A,b^	42.1 ± 1.6^A,cd^	3237.7 ± 164.6^A,ab^	2615.8 ± 48.1^A,a^
PC12(Annealed)	29.0 ± 2.1^A^	36.0 ± 1.5^A,b^	41.7 ± 0.5^A,d^	2725.4 ± 592.5^AB,b^	2140.1 ± 406.5^AB,ad^

a Storage modulus values at −20 and 23 °C have also been reported. In all cases, the glass transition temperature increased in the order of: *T*_g_ at onset of *E*′ (°C) < *T*_g_ at *E*″ peak (°C) < *T*_g_ at tan δ peak (°C).

b Data are presented as mean ± standard deviation (*n* = 3–6). Means lacking a shared letter within the same column indicate statistically significant differences at *P*-value < 0.05, as determined by Tukey’s test. Uppercase letters, such as “A”, signify significant differences within a specific group, whereas lowercase letters, such as “a”, denote significant differences between groups.

For *T*_g_ determined from the peak of tan δ, the analysis indicated that only the treatment had a statistically significant effect on *T*_g_ (*F*-value = 28.29, *P*-value < 0.001). These results indicate that the changes in *T*_g_ between the groups (Table 3) were mainly associated with the treatment conditions rather than differences in the sample formulation. Statistical analysis showed no difference in *T*_g_ within each group, whereas the annealed samples (group 4) were significantly different from the other groups. Upon examining the interaction between the sample formulation and treatment, the highest *T*_g_ values were recorded for the PVAc samples following heat treatment (groups 2 and 3; approximately 53 °C). In contrast, the lowest *T*_g_ values were observed for the annealed nanocomposites (group 4), particularly for PC9 and PC12, which exhibited values of 42.1 and 41.7 °C, respectively, with a significant difference (Table 3). No significant difference in *T*_g_ was observed among the PVAc samples across the four groups, suggesting that heat treatment did not lead to a notable variation in *T*_g_ for PVAc samples. Among the nanocomposites, PC12 exhibited the largest response to annealing, showing an approximately 8 °C (16%) reduction in *T*_g_ relative to untreated PC12, suggesting a structural rearrangement within the PVAc/CNCs network during thermal treatment. A decrease in *T*_g_ has been observed in nanocomposites characterized by weak interfacial interactions.^44^ If the observed decrease in the glass transition temperature is attributed to the transport of free-volume holes within the bulk of the nanocomposites, rather than to increased chain mobility, then the glass transition may be considered to occur when these free-volume holes are capable of diffusing and redistributing throughout the material.^44^ However, the validation of this hypothesis requires more advanced methodologies for examining the free volume of polymers, specifically through positron annihilation lifetime spectroscopy (PALS).^45^ An alternative explanation for the observed decrease in *T*_g_ can be inferred from polarization microscopy studies, which reveal alterations in the topographical structure after annealing (Figure 3e,f). It has been reported that nanocomposites with higher CNCs concentrations exhibit increased topographical structures. This structure is reduced following annealing, which imposes constraints on the polymer network, as evidenced by the study conducted by Lee and Lichtenhan.^39^

A general linear model (GLM) was employed to assess the effects of the formulation, treatment, and their interaction on *T*_g_, as determined from the onset of the storage modulus decline. Owing to the presence of heteroscedasticity, as indicated by residual diagnostics, a Box–Cox transformation was applied prior to the analysis. The results indicated that the treatment exerted a statistically significant effect on *T*_g_ (*P*-value = 0.028), whereas neither the formulation nor the interaction term demonstrated a significant impact. However, Tukey’s pairwise comparisons failed to identify significant differences among the treatment means, likely due to experimental variability and relatively minor differences between the group means.

A similar analysis was performed for the *T*_g_ determined from the *E*″ loss modulus peak. The GLM results indicated that treatment had a significant effect on *T*_g_ (*P*-value < 0.001), whereas sample formulation and the interaction between formulation and treatment were not statistically significant. Tukey pairwise comparisons indicated no statistically significant difference between the annealed and untreated samples. However, a significant difference was observed when the annealed samples (group 4) were compared with the heat-treated samples (groups 2 and 3), suggesting that annealing decreased the glass transition temperature, as determined from the loss modulus peak (Figure 9b4).

The modulus quantifies a material’s resistance to deformation under the application of an external force. The storage modulus *E*′ reflects the elastic response of the material; thus, a higher *E*′ denotes increased material stiffness and resistance to deformation. As amorphous polymers are generally isotropic, the integration of anisotropic cellulose nanocrystals may induce direction-dependent behavior. At −20 °C, the samples exhibited a high storage modulus, indicating that they were in a glassy state and were stiff. As the temperature increased, the storage modulus decreased, particularly with the onset of the glass transition temperature. Figure 9c demonstrates that an increase in the CNCs content enhanced the stiffness of the nanocomposite samples beyond that of PVAc. Furthermore, heat treatment contributed to an additional increase in stiffness.

At −20 °C, PVAc exhibited the highest storage modulus in group 1, significantly exceeding the values of the other samples in the group. While no differences were observed among the samples after 500 h of heat treatment (group 2), significant differences were identified between PVAc and PC9 within two other groups (3 and 4).

Upon comparing the storage moduli among the groups at −20 °C, it was observed that the storage modulus was primarily influenced by the applied treatment (*P*-value = 0.043) and its interaction with the formulation (*P*-value = 0.001), while the effect of the formulation alone was not statistically significant (*P*-value = 0.28). The highest storage modulus was observed for annealed PC9 (∼3238 MPa), followed by untreated PVAc (∼3106 MPa), with no significant difference between the two. In contrast, the lowest storage modulus was measured for annealed PVAc (∼1986 MPa), which was significantly lower than that of annealed PC9. Annealing resulted in an increase in the storage modulus at −20 °C for all the nanocomposite samples, with no significant differences observed among them. In contrast, it led to a substantial decrease in the modulus of PVAc, with the average value declining by approximately 36%.

At 23 °C, PVAc and PC12 in group 1 demonstrated the highest storage modulus, with no notable difference between them. Conversely, for the annealed samples (group 4), a significant difference was observed between PC9 and PVAc. The results demonstrated that annealing resulted in either an increase or no detrimental effect on the storage modulus of the nanocomposites, depending on the CNCs concentration, whereas a decrease was observed for pristine PVAc. No discernible changes in the storage modulus were observed for the heat-treated samples (groups 2 and 3), suggesting that the heat treatment did not compromise the mechanical durability of the samples. When aging causes a decrease in the material’s free volume, the polymer structure is anticipated to reach a more stable condition, which generally leads to an increase in the storage modulus.^26^ However, the present study did not observe such an increase, suggesting that the aging process did not significantly reduce the free volume of the material under the investigated conditions.

A creep test was used to evaluate the response of a material to a constant load and its behavior after the removal of that load. A general linear model was applied to evaluate the effects of sample formulation, treatment, and the interaction between formulation and treatment on the maximum strain, creep percentage, and creep compliance. The ANOVA results demonstrated that all factors (formulation, treatment, and interaction between them) significantly affected the maximum strain (*P*-value < 0.001). Among these factors, sample formulation exhibited the largest effect (*F*-value = 70), whereas treatment and the interaction between formulation and treatment showed smaller effects with *F*-values of 21 and 5, respectively.

Figure 10a illustrates that an increase in the CNCs content resulted in a progressively flatter equilibrium region, signifying that the nanocomposites attained their maximum strain and reached a stable deformation state more rapidly. In contrast, PVAc demonstrated a continuous increase in strain over time under nearly all conditions, except for the 500 h heat treatment, suggesting that equilibrium was not achieved within the experimental time frame.

**Figure 10:**
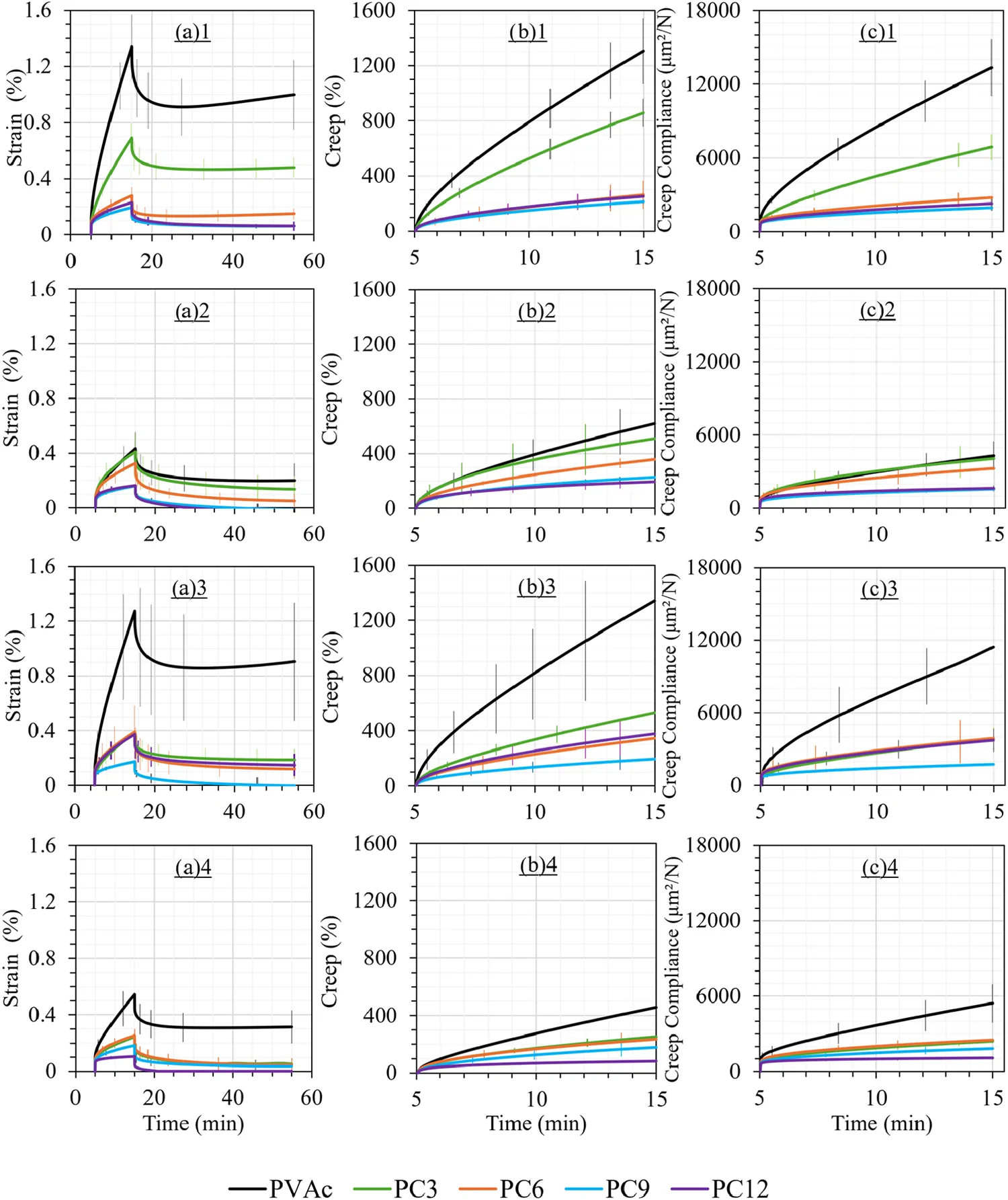
(a) Strain %, (b) creep %, and (c) creep compliance (μm^2^/N) of PVAc and nanocomposites: (1) untreated samples, (2) samples after 500 h of heat treatment, (3) samples after 1000 h of heat treatment, and (4) annealed samples.

Among the different treatments, annealing and 500 h heat treatment showed the strongest influence on the maximum strain, although no statistically significant difference was observed between them. The highest maximum strain was measured for pristine PVAc (∼1.3%), indicating the greatest susceptibility to deformation. The findings indicate a progressive decrease in the maximum strain with increasing CNCs content in the nanocomposites (Table 4). The annealed PC12 exhibited the lowest strain, measured at approximately 0.1%, which was significantly different from that of the other samples. This constitutes a reduction of approximately 92% compared to pristine PVAc and 54% to the untreated PC12. The observed reduction in the maximum strain following 500 h of aging and annealing indicates structural collapse and densification. This process involves the gradual rearrangement of polymer chains toward a more stable configuration, thereby reducing chain mobility, which consequently increases the brittleness of the material. The increased compactness of the material likely enhances the hydrogen bonding between the ester groups on the polymer chains and the hydroxyl groups on the CNCs. In the nanocomposites, the influence of the CNCs on limiting the segmental motion became more pronounced after 500 h of aging and annealing treatment. The 1000 h heat treatment did not yield results comparable to those of the other heat treatments, particularly in the case of PVAc.

**Table 4:** Maximum Strain %, Maximum Strain Recovery %, Maximum Creep %, and Maximum Creep Compliance (μm^2^/N) for Untreated Samples (Group 1), Samples after 500 h Heat Treatment (Group 2), Samples after 1000 h Heat Treatment (Group 3), and Annealed Samples (Group 4)^a^

Sample	Max Strain %	Max Strain Recovery %	Max Creep %	Max Creep Compliance (μm^2^/N)
Untreated samples (group 1)				
PVAc	1.3 ± 0.2^A,a^	32.7 ± 6.3^C^	1304 ± 239.8^A,a^	13336.1 ± 2301.5^A,a^
PC3	0.7 ± 0.1^B,a^	34.2 ± 2.2^C^	858.4 ± 101.3^B,ab^	6880 ± 1060^B^
PC6	0.3 ± 0.0^BC^	53.3 ± 8.3^B^	264.9 ± 102.3^C^	2777.7 ± 608.7^BC^
PC9	0.2 ± 0.0^C^	69.2 ± 6.9^AB^	213.3 ± 10.9^C^	1947.5 ± 78^C^
PC12	0.2 ± 0.0^C^	74.4 ± 8.8^A^	252.3 ± 70.8^C^	2282.4 ± 552.5^C^
Samples after 500 h heat treatment (group 2)				
PVAc-500h	0.4 ± 0.1^A^	58.6 ± 18.5^C^	619.6 ± 186.2^A,b^	4312.9 ± 1192.6^A^
PC3–500h	0.4 ± 0.1^A^	71.1 ± 19.3^BC^	509.3 ± 202.7^AB^	4073.9 ± 1381.2^A^
PC6–500h	0.3 ± 0.0^AB^	86.1 ± 13.9^ABC^	359.2 ± 40.3^AB^	3256.0 ± 782.3^AB^
PC9–500h	0.2 ± 0.0^B^	111.5 ± 27.4^AB^	227.1 ± 23.2^B^	1580.9 ± 227.6^B,h^
PC12–500h	0.2 ± 0.0^B^	121.2 ± 16.1^A^	195.3 ± 41.6^B^	1638.6 ± 256.7^B,h^
Samples after 1000 h heat treatment (group 3)				
PVAc-1000h	1.0 ± 0.5^A,a^	33.9 ± 7.2^B^	1342.4 ± 575.2^A,ab^	12701.7 ± 5057.4^A,a^
PC3–1000h	0.4 ± 0.1^B^	52.9 ± 8.8^B^	529.8 ± 101.8^B^	3870.7 ± 958.1^B^
PC6–1000h	0.4 ± 0.2^B^	70.6 ± 11.3^AB^	344.9 ± 109.1^B^	3886.8 ± 1918.2^B^
PC9–1000h	0.2 ± 0.0^B^	104.6 ± 36.2^A^	194.6 ± 71.6^B^	1738.1 ± 121.6^B^
PC12–1000h	0.3 ± 0.1^B^	61.0 ± 15.6^AB^	377.4 ± 138.4^B^	3729.0 ± 958.3^B^
Annealed samples (group 4)				
PVAc (Annealed)	0.5 ± 0.1^A,a^	44.4 ± 4.5^B^	456.2 ± 11.8^A,g^	5427.9 ± 1523.2^A^
PC3 (Annealed)	0.2 ± 0.0^B^	84.3 ± 11.2^B^	253.6 ± 36.4^B,gh^	2423.8 ± 251.6^B^
PC6 (Annealed)	0.2 ± 0.0^B^	83.2 ± 12.9^B^	236.8 ± 66.6^B,gh^	2523.9 ± 469.9^B^
PC9 (Annealed)	0.2 ± 0.0^BC^	84.3 ± 27.8^B^	177.6 ± 56.2^B,h^	1841.7 ± 394.7^BC^
PC12 (Annealed)	0.1 ± 0.0^C,g^	121.8 ± 10.4^A^	82.7 ± 11.1^C,i^	1068.7 ± 136.7^C,h^

a Data are presented as mean ± standard deviation (*n* = 3–6). Means lacking a shared letter within the same column indicate statistically significant differences at *p* < 0.05, as determined by Tukey’s test. Uppercase letters, such as “A”, signify significant differences within a specific group, whereas lowercase letters, such as “a”, denote significant differences between groups.

The findings indicated that all factors (formulation and heat treatment) and their interactions exerted a significant influence on creep, with the sample formulation demonstrating the most pronounced effect (*P* = 0.000, *F* = 63). Among the various heat treatment methods, annealing significantly reduced creep compared to the untreated samples or samples subjected to alternative heat treatments.

For the untreated samples, pristine PVAc demonstrated the highest creep, whereas PC9 and PC12 exhibited the lowest values, with no significant difference observed between them. A study conducted by Ataei, Mohammadi^46^ indicated that inadequate dispersion of CNCs results in agglomeration, which induces stress concentration and consequently reduces creep performance. As the highest concentration of CNCs in untreated PC12 did not reduce the creep performance, it can be suggested that the degree of aggregation was not excessively high. The interaction analysis indicated that the most pronounced reduction in creep was observed when high CNCs loading was combined with annealing. The combined effect of annealing and the highest CNCs loading decreased the mean creep of PC12 by approximately 93% compared to pristine PVAc, indicating a substantial improvement in the resistance to time-dependent deformation and improved long-term durability. In the present study, as all samples were subjected to identical stress conditions, both creep and creep compliance exhibited consistent trends.

Understanding the creep behavior of polymer films provides insight into their long-term mechanical stability and functional performance. Lower creep generally indicates a higher resistance to time-dependent deformation, which is beneficial for maintaining dimensional stability and preserving the original coating morphology. In coating applications, this behavior contributes to coating uniformity, allowing the coating layer to retain its thickness and structural integrity during the service. Reduced deformation also enhances mechanical durability, as the coating better resists permanent deformation under pressure, bending, and stacking loads. Moreover, limited creep is beneficial for maintaining adhesion stability, as excessive deformation can create stress at the coating–substrate interface, potentially resulting in delamination. From a barrier perspective, the reduction in creep is advantageous because substantial viscoelastic deformation can induce microstructural alterations in the film, potentially increasing the pathways for water vapor transmission. Conversely, coatings with enhanced creep properties may confer specific functional advantages depending on their intended application. Enhanced viscoelastic deformation promotes stress relaxation, enabling the material to dissipate internal stress, thereby reducing the likelihood of crack formation. This characteristic can also enhance adaptability, which may be advantageous when the coated surface experiences repeated bending or folding, as observed in flexible packaging or materials made from paper. Thus, achieving ideal creep behavior involves finding a balance between maintaining dimensional stability and barrier performance, while ensuring the flexibility required for specific applications.

### 3.10 Tensile Properties

The tensile modulus, tensile strength, and elongation at break were statistically analyzed using a general linear model to evaluate the effects of the formulation, tensile speed, and annealing, including their interaction terms. All untreated samples were tested at two tensile speeds (1 and 5 mm·min^–1^) (Figure 11a,c, respectively). After annealing, only the nanocomposites were tested and analyzed at two tensile speeds (1 and 5 mm·min^–1^) (Figure 11b,d).

**Figure 11:**
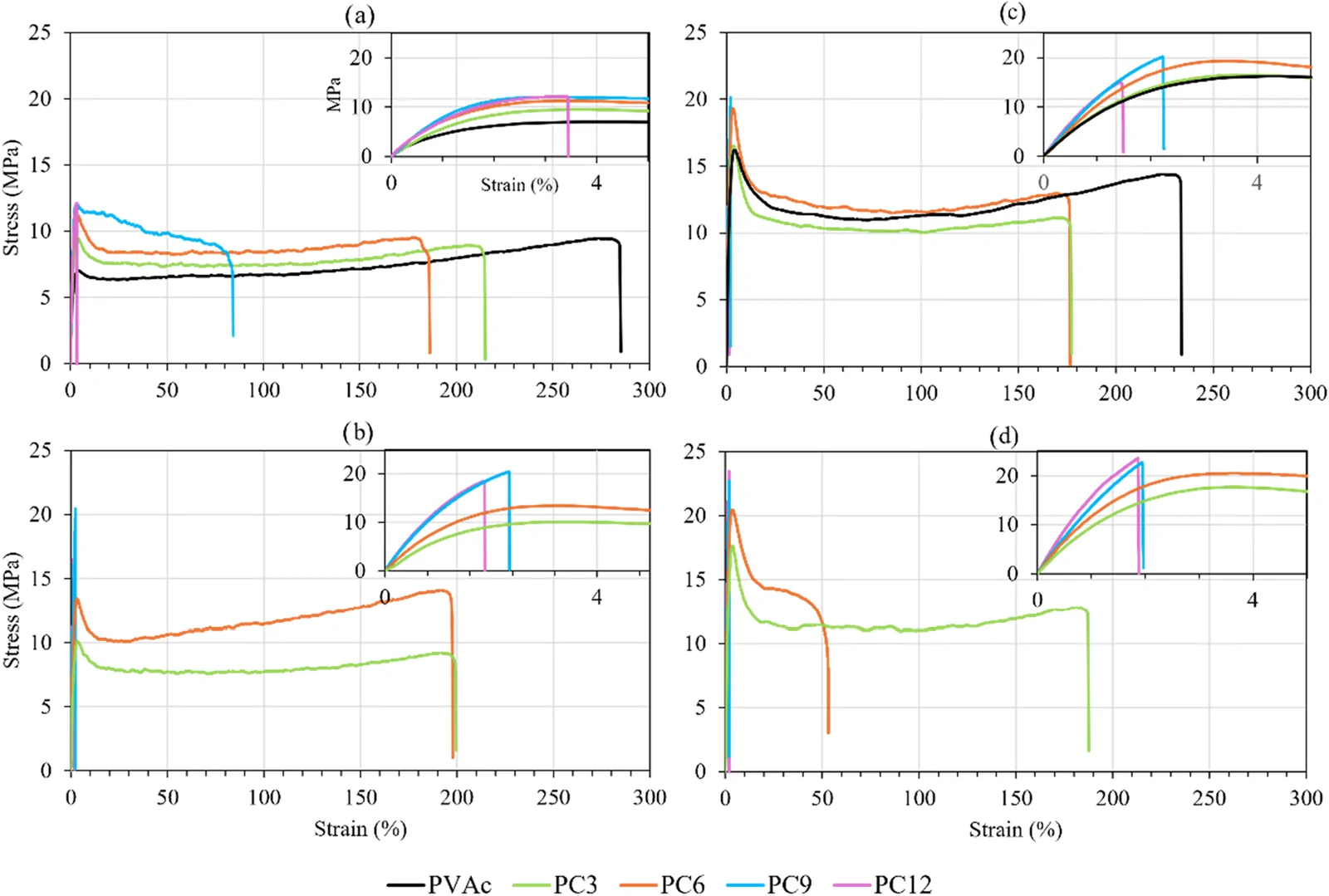
Stress–strain curves of the samples obtained from tensile testing: (a) untreated samples tested at a crosshead speed of 1 mm·min^–1^, (b) annealed samples tested at 1 mm·min^–1^, (c) untreated samples tested at 5 mm·min^–1^, and (d) annealed samples tested at 5 mm·min^–1^. Only the nanocomposite samples were tested after annealing.

#### 3.10.1 Effect of Formulation, Annealing, and Speed on Tensile Modulus

In the untreated samples, including pristine PVAc, formulation was the dominant factor influencing elastic modulus, accounting for 49% of the total variance (*P* = 0.001), followed by interaction between formulation and speed (15.4%) and tensile speed (10.9%). Pristine PVAc tested at a speed of 1 mm/s demonstrated the lowest modulus (∼448 MPa), which was significantly different from that of all other samples. Although an increase in the testing speed resulted in a statistically significant difference in the modulus for PC3 and PC6, no significant effect was observed for the other nanocomposites. The most significant increase in modulus was observed at a testing speed of 1 mm/min for PC12, which was 212% greater than that of PVAc.

Statistical analysis demonstrated that all factors, including formulation (31.8%), tensile speed (8.9%), and annealing (14%), as well as their interactions with the sample, significantly influenced the nanocomposite modulus (*P* < 0.005). Interaction analysis demonstrated that the annealing effect was formulation-dependent, with the largest improvement observed for formulations with high CNCs content (PC9 and PC12). The maximum modulus among the samples was observed for PC9 at a testing speed of 1 mm·min^–1^ following annealing, reaching 2043 MPa, with no significant difference observed at higher testing speeds. The annealing process significantly affected the elastic modulus of PC9, resulting in an increase of 95% at speed 1 and 60% at speed 5 mm/min. The highest recorded modulus of annealed PC9 represents a 356% increase compared to that of the untreated PVAc tested at the same speed.

#### 3.10.2 Effect of Formulation, Annealing, and Speed on Ultimate Tensile Strength

Statistical analysis of the untreated samples demonstrated that the formulation, tensile speed, and their interaction had a significant effect on tensile strength (*P* < 0.005). Tensile speed was the most influential factor, accounting for 45.5% of the total variance, followed by the formulation (25.1%). The interaction effect was comparatively lower, contributing to 9% of the total variance. The highest tensile strength was observed for PC9 and PC12 at a testing speed of 1 mm/min, which was significantly higher than those of PVAc and PC3. The maximum ultimate strength among the untreated samples was observed for PC6 (19.7 MPa), which was 52.7% higher than that of PVAc (12.9 MPa) at a testing speed of 5 mm·min^–1^.

The comparative analysis of the nanocomposites revealed that the incorporation of annealing rendered all factors, including formulation, testing speed, and annealing, as well as the interactions among these factors, statistically significant (*p* < 0.005). The tensile speed was identified as the most influential parameter, accounting for 31.2% of the total variance. This was followed by formulation, which contributed 23.5%, and annealing, which accounted for 13.6% of the variance. Following annealing, no significant difference was observed in the tensile strength of the annealed nanocomposites, regardless of whether they were tested at 1 or 5 mm/min. At a testing speed of 1 mm/min, annealing resulted in an increase in the tensile strength of approximately 49% for PC6 (from 17.1 to 11.5 MPa) and 39% for PC9 (from 20.3 to 14.6 MPa). At a testing speed of 5 mm/min, while PC12 exhibited a 54% increase postannealing, no significant difference was noted in the tensile strength of the other nanocomposites. The maximum ultimate tensile strength was 23.3 MPa for the annealed PC12 at 5 mm min^–1^, representing an 80% increase compared with that of pristine PVAc (12.9 MPa) tested at 5 mm min^–1^.

#### 3.10.3 Effect of Formulation, Annealing, and Speed on Elongation at Break

The statistical analysis of untreated samples showed that all factors and interactions had a significant effect on the elongation at break, with the most prominent contribution from the formulation (89.4%). The results showed that the elongation at break decreased with increasing CNCs concentration. The effect of a higher speed generally decreased the mean elongation of untreated samples (Figure 11c). The maximum elongation was observed for untreated PVAc at a tensile speed of 1 mm/min, reaching approximately 280% elongation. Notably, increasing the tensile speed did not significantly influence the measured value. Among the untreated nanocomposites, PC3 and PC6 exhibited no significant variation in elongation when the testing speed was increased from 1 to 5 mm/min. Following the annealing process, all factors and their interactions exhibited a significant impact on the elongation of the nanocomposites, while the formulation accounted for 87.6% of the total variance. The findings indicate that PC3 and PC6 retained their ductility and exhibited consistent elongation values following annealing at a testing speed of 1 mm/min, whereas the annealing rendered other nanocomposites brittle. Upon testing the annealed samples at an increased speed of 5 mm/min, PC3 maintained its ductility without significant alteration, whereas PC6 experienced a notable reduction in ductility.

The findings indicate that an increased incorporation of CNCs results in an enhanced modulus. However, if the concentration becomes excessively high, leading to aggregation or agglomeration, it negatively impacts the material properties, which is consistent with previous studies.^47^ The PC12 and PC9 samples exhibited a higher propensity for CNCs aggregation, as illustrated in Figure 3. The greater elongation observed in PC9 may suggest lower aggregation and fewer stress concentrators.

### 3.11 Thermogravimetric Analysis

Figure 12 shows the TGA curves of PVAc and the nanocomposites. As illustrated, the incorporation of CNCs increased the degradation temperature of the nanocomposites. For PVAc, the rate of mass loss was greater than that observed for the nanocomposites, as illustrated in Figure 12B. The findings indicated that the incorporation of CNCs delayed the degradation of the nanocomposites compared to pristine PVAc. Degradation in all samples occurred in two distinct stages, with the most significant degradation observed in the first stage, accounting for approximately 65%, whereas the second stage contributed approximately 25% to the degradation. The reduced degradation observed in the initial stage of the nanocomposite may be attributed to the interactions between the ester bonds in PVAc and the hydroxyl groups in the cellulose nanocrystals. These interactions potentially delay the release of acetic acid, which has been previously identified as a factor contributing to degradation during the initial stage.^48,49^ The degradation of CNCs also contributed to the observations shown in Figure 12. Without proper connections, the CNCs degraded more rapidly than when they were incorporated into the nanocomposites, which may account for the observed delay in degradation within the nanocomposites. The interfacial interactions between the CNCs and polyvinyl acetate matrix are crucial for maintaining their thermal stability. The elevated onset of degradation indicates that the CNCs are effectively integrated and dispersed through the formation of chemical bonds.^50^ This close association fosters a more stable structure, although its primary influence is observed in the mechanical properties rather than in the thermal properties. Figure 12a further illustrates that the enhancement in thermal stability achieved through the incorporation of CNCs does not exhibit a significant variation with different CNCs concentrations.

**Figure 12:**
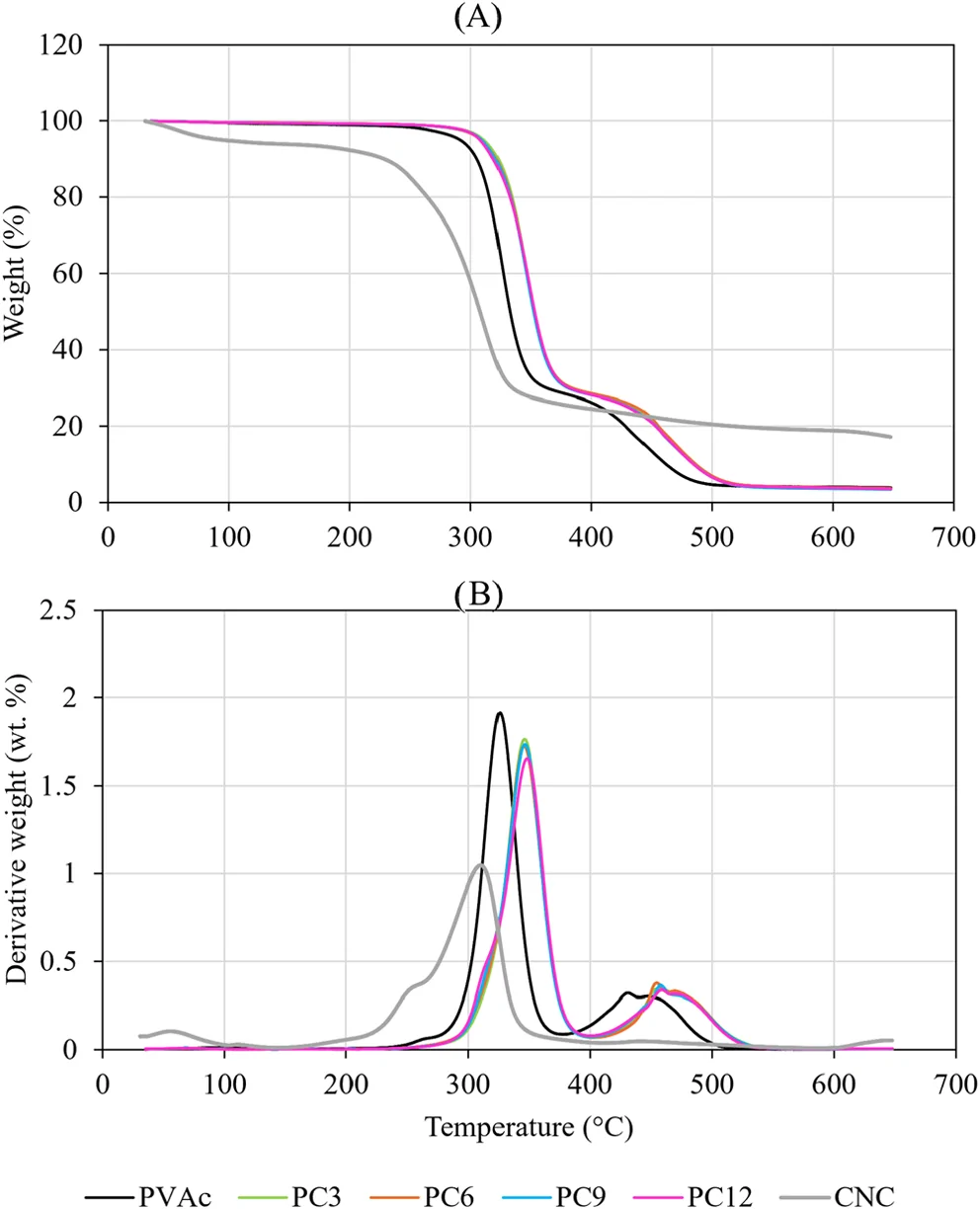
TGA curves of PVAc and nanocomposites (A) weight loss versus temperature, (B) derivative weight changes by the temperature.

At low weight-loss levels (2–5%), the nanocomposites exhibited consistently higher onset temperatures than PVAc, confirming the stabilizing effect of the CNCs during the initial degradation stage. At 50% weight loss, the nanocomposite samples exhibited degradation temperatures in the range of 339–340 °C, which is slightly higher than that of PVAc (330.4 °C). The char yields of the nanocomposites were slightly lower than that of PVAc, indicating that the addition of CNCs did not increase the char yield relative to that of PVAc.

## 4 Conclusions

This study investigated the effects of different parameters, including CNCs concentration, low reaction temperature, surfactants, and various heat treatments, on PVAc/CNCs nanocomposites synthesized at a total solid content of 48 to 49%. Dynamic mechanical analysis revealed that the incorporation of CNCs did not affect the glass transition temperature. However, after annealing, the *T*_g_ of the nanocomposites decreased, especially for the highest CNCs loading (2.5 wt % CNCs/VAc; PC12). This could be attributed to the presence of CNCs aggregates scattered between the polymer chains. These unconnected agglomerates caused partial phase separation by preventing chain packing. The results showed that annealing decreased the *T*_g_ of PC12 by approximately 8 °C. The *T*_g_ values of the samples differed significantly only when measured from the peak of the loss modulus, and this difference was observed exclusively between the heat-treated and annealed samples. The findings suggest that heat treatment did not adversely affect the mechanical durability of the samples within the investigated time frame, as evidenced by the storage modulus results.

According to the results, annealing decreased the width of tan δ for the nanocomposites with 0.6 and 1.2 wt % of CNCs/VAc. Consequently, the reduction in the breadth of tan δ indicates lower heterogeneity within the sample after annealing. This was further verified by the light transmittance results, which showed that annealed PC3 and PC6 had higher light transmittance, indicating fewer areas available for light scattering. The enhanced interaction between the cellulose nanocrystals and polymer matrix was further corroborated by water contact angle measurements. Although no significant differences were observed among the untreated samples, annealing increased the contact angle, particularly at higher CNCs loadings. This suggests that the nanocomposite structure became more robust and exhibited reduced polarity, leading to a decrease in hydrophilicity. The integration of a moderate quantity of CNCs particles into the polymer matrix led to a significant reduction in water absorption and enhancement of structural integrity (sample PC3), as demonstrated by the swelling tests. Nevertheless, the effect of annealing was only significant on the swelling percentage of nanocomposites with elevated CNCs content. All the nanocomposites demonstrated reduced permeability relative to that of pristine poly­(vinyl acetate). Among the untreated samples, the nanocomposites with the highest concentration of cellulose nanocrystals exhibited the lowest permeability. The annealing process solely influenced the permeability of the nanocomposite with the lowest CNCs concentration (sample PC3), resulting in values comparable to those of the samples with the highest CNCs loading. This behavior can be attributed to the reduced presence of CNCs particles dispersed among the polymer chains, which permits better modification after annealing. The results showed that lower CNCs loading facilitated effective annealing-induced structural rearrangements, improved filler–matrix interactions, and reduced film nonuniformities.

The study showed that CNCs can help the material resist the reduction in storage modulus at higher temperatures, whereas pristine PVAc experienced a significant reduction. The creep tests on the samples showed that the combined effect of increasing the CNCs concentration and annealing had the highest influence on reducing the maximum strain. Therefore, the annealed PC12 samples demonstrated the lowest creep among the samples. Statistical analysis demonstrated that both the CNCs concentration and annealing process exerted a significant influence on the modulus, ultimate strength, and elongation at break. The highest modulus among the untreated samples was observed for PC12, with the highest CNCs loading, which was approximately 212% higher than that of untreated PVAc. After annealing, the highest recorded modulus was for annealed PC9, which was 356% higher than that of untreated PVAc. The tensile strength was primarily affected by formulation, and annealing did not have any influence. The elongation properties of the nanocomposites were significantly influenced by the loading of CNCs, with a higher CNCs content resulting in reduced elongation. Notably, the nanocomposite PC3 with a CNCs/VAc concentration of 0.6 wt % maintained its elongation capacity even after annealing or when the tensile speed was increased from 1 to 5 mm/min. In contrast, other nanocomposites with higher CNCs loadings were unable to preserve ductility.

## Data Availability

The data are available from the corresponding author upon reasonable request.
